# Anaerobic endosymbiont generates energy for ciliate host by denitrification

**DOI:** 10.1038/s41586-021-03297-6

**Published:** 2021-03-03

**Authors:** Jon S. Graf, Sina Schorn, Katharina Kitzinger, Soeren Ahmerkamp, Christian Woehle, Bruno Huettel, Carsten J. Schubert, Marcel M. M. Kuypers, Jana Milucka

**Affiliations:** 1 Max Planck Institute for Marine Microbiology, Bremen, Germany; 2 Division of Microbial Ecology, Centre for Microbiology and Environmental Systems Science, University of Vienna, Vienna, Austria; 3 Max Planck Genome Centre Cologne, Max Planck Institute for Plant Breeding Research, Cologne, Germany; 4 Surface Waters - Research and Management, Eawag, Kastanienbaum, Switzerland

**Keywords:** Microbial ecology, Evolution, Microbial genetics, Symbiosis, Element cycles

## Abstract

Mitochondria are specialized eukaryotic organelles that have a dedicated function in oxygen respiration and energy production. They evolved about 2 billion years ago from a free-living bacterial ancestor (probably an alphaproteobacterium), in a process known as endosymbiosis^1,2^. Many unicellular eukaryotes have since adapted to life in anoxic habitats and their mitochondria have undergone further reductive evolution^3^. As a result, obligate anaerobic eukaryotes with mitochondrial remnants derive their energy mostly from fermentation^4^. Here we describe ‘*Candidatus* Azoamicus ciliaticola’, which is an obligate endosymbiont of an anaerobic ciliate and has a dedicated role in respiration and providing energy for its eukaryotic host. ‘*Candidatus* A. ciliaticola’ contains a highly reduced 0.29-Mb genome that encodes core genes for central information processing, the electron transport chain, a truncated tricarboxylic acid cycle, ATP generation and iron–sulfur cluster biosynthesis. The genome encodes a respiratory denitrification pathway instead of aerobic terminal oxidases, which enables its host to breathe nitrate instead of oxygen. ‘*Candidatus* A. ciliaticola’ and its ciliate host represent an example of a symbiosis that is based on the transfer of energy in the form of ATP, rather than nutrition. This discovery raises the possibility that eukaryotes with mitochondrial remnants may secondarily acquire energy-providing endosymbionts to complement or replace functions of their mitochondria.

## Abstract (web summary)

‘*Candidatus* Azoamicus ciliaticola’ transfers energy to its ciliate host in the form of ATP and enables this host to breathe nitrate, demonstrating that eukaryotes with remnant mitochondria can secondarily acquire energy-providing endosymbionts.

## Main

Eukaryotic life evolved at least around 1–1.9 billion years ago^5,6^, after the gradual oxygenation of the atmosphere of the Earth (which started about 2.4 billion years ago^7,8^). The ensuing diversification of eukaryotes has been attributed to their ability to harvest copious amounts of energy from the respiration of oxygen, an abundant, high-potential electron acceptor. Oxygen respiration is performed in mitochondria, which are specialized subcellular organelles that arose from a free-living prokaryotic organism through endosymbiosis^9^. During the evolution towards the contemporary organelle, the endosymbiont has either lost its genes or transferred them to the host genome; mitochondria have kept only a small subset of genes^10^ that pertain to translation and the electron transport chain^11,12^.

Some eukaryotes that inhabit anoxic environments do not contain aerobic mitochondria. Originally thought to be ‘ancestral’^13^, all of these amitochondriate eukaryotes in fact evolved secondarily from predecessors that had aerobic mitochondria^14^; they possess highly reduced mitochondria-related organelles or at least contain genes of mitochondrial origin in their nuclear genomes^15^. Throughout the course of evolution, anaerobic unicellular protists have lost the capacity to generate ATP via oxidative phosphorylation (with some exceptions^16,17^). Instead, these eukaryotes generate ATP from fermentation through substrate-level phosphorylation, coupled to the formation of H_2_ (ref. ^18^). This reaction takes place in the cytoplasm or—more commonly—in hydrogenosomes. Hydrogenosomes are specialized organelles, which have independently evolved from mitochondria in evolutionarily distant organisms^18,19^. Some ciliates that contain hydrogenosomes also contain endosymbiotic methanogenic archaea that scavenge the produced H_2_, which thereby increases the energy yield of the reaction^20^.

In contrast to strict anaerobes that exclusively ferment, some facultatively anaerobic eukaryotes can perform anaerobic respiration^21^. Nitrate and/or nitrite reduction has previously been demonstrated for some foraminifera and Gromiida^22–24^, fungi^25,26^ and the ciliate *Loxodes*^27^. The underlying molecular mechanism of eukaryotic denitrification and energy conservation is largely unknown. Here we report the discovery of an obligate endosymbiont of an anaerobic ciliate that provides its host with energy derived from denitrification.

## Anaerobic ciliates with bacterial endosymbionts

**Extended Data Fig. 1:**
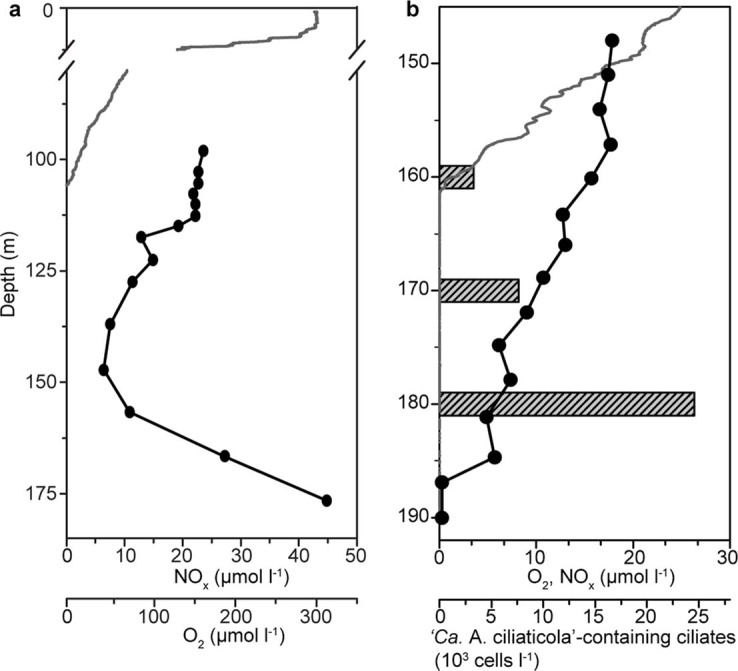
Lake Zug physicochemical profiles and abundance of ciliates containing ‘*Ca*. A. ciliaticola’. **a**, **b**, Depth profiles of oxygen and NO_*x*_ (nitrate + nitrite) throughout the lower water column of Lake Zug measured in 2016 (**a**) and 2018 (**b**). In 2018, the abundance of ciliates containing ‘*Ca*. A. ciliaticola’ (shown as grey bars in **b**) from immediately below the base of the oxycline (160 m) was 3.2 × 10^3^ cells per litre (*n* = 29 cells), which corresponds to approximately half of all unicellular eukaryotes at this depth. At 180 m, all investigated eukaryotes were ciliates that contain ‘*Ca*. A. ciliaticola’, and their abundance had increased to 24.5 × 10^3^ cells per litre (*n* = 77 cells) (Supplementary Table 1). Source data

We investigated Lake Zug, an approximately 200-m deep and permanently stratified freshwater lake that is located in Switzerland. Lake Zug contains deep waters that remain permanently anoxic and replete in nitrate, which is a feature that is characteristic of many stratified fresh and marine waters depleted in oxygen. The thickness of the anoxic hypolimnion varied between 40 and 100 m over a period of 5 years (2016—2020) (Extended Data Fig. 1). In 2018, oxygen was undetectable below a depth of 160 m, and a decrease in NO_*x*_ (mainly nitrate) concentration below the base of the oxycline indicated ongoing denitrification (Extended Data Fig. 1).

**Fig. 1:**
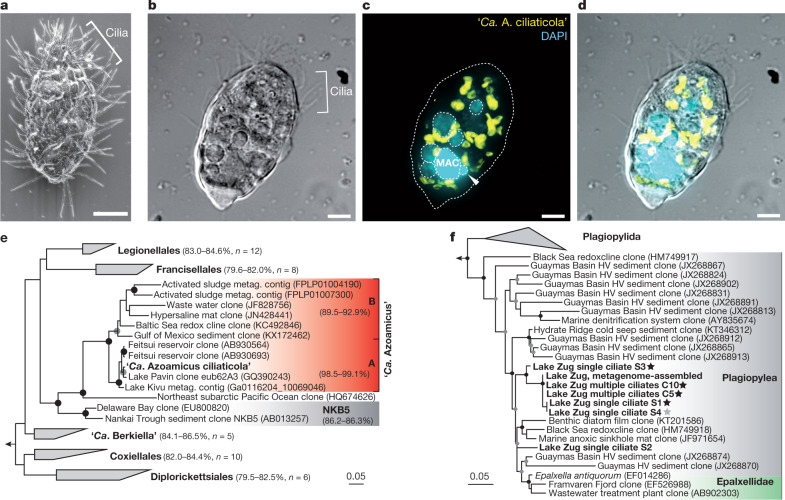
Visualization and phylogenetic affiliation of ‘*Ca*. A. ciliaticola’ and its ciliate host. **a**, Representative scanning electron microscopy image of a ciliate from the anoxic hypolimnion of Lake Zug (February 2020, depth of 186 m). Scale bar, 5 μm. **b**–**d**, Differential interference contrast image (**b**) and confocal laser scanning microscopy image (**c**) of a ciliate after hybridization with a ‘*Ca*. Azoamicus’-specific oligonucleotide probe (eub62A3_813) (yellow) and counterstaining with DAPI (blue). **d**, Overlay of fluorescence and differential interference contrast images. The macronucleus (MAC) with attached micronucleus (arrowhead) and putative food vacuoles of the ciliate are outlined. Scale bars, 5 μm. **e**, 16S rRNA gene sequence-based maximum likelihood phylogenetic tree of ‘*Ca*. A. ciliaticola’ (bold denotes sequence derived from circular metagenome-assembled genome) and members of related gammaproteobacterial orders and environmental clades. Subgroups A and B of the ‘*Ca*. Azoamicus’ group are indicated. The sequence similarities of respective groups to the 16S rRNA gene sequence of ‘*Ca*. A. ciliaticola’ are shown in parentheses. The full tree is shown in Extended Data Fig. 4. Taxonomic groups were assigned on the basis of SILVA taxonomy^45^. Metag., metagenome. **f**, Ciliate 18S rRNA gene sequence-based maximum likelihood phylogenetic tree of the class Plagiopylea based on EukRef-Ciliophora reference alignment^30^. Sequences of ciliates from Lake Zug (in bold) were amplified from individual ciliates (S1–S4) or combined ciliates (C5 and C10). Stars denote positive identification of a ‘*Ca*. A. ciliaticola’ 16S rRNA sequence amplified from the same ciliate: black, ‘*Ca*. A. ciliaticola’ 16S rRNA gene sequence verified by Sanger sequencing; grey, positive band on gel. For both trees, bootstrap values are shown as circles at the respective nodes, and indicate bootstrap support of >70% (grey) or >90% (black) out of 100 resamplings. Scale bars, 0.05 nucleotide substitutions per site.

**Extended Data Fig. 2:**
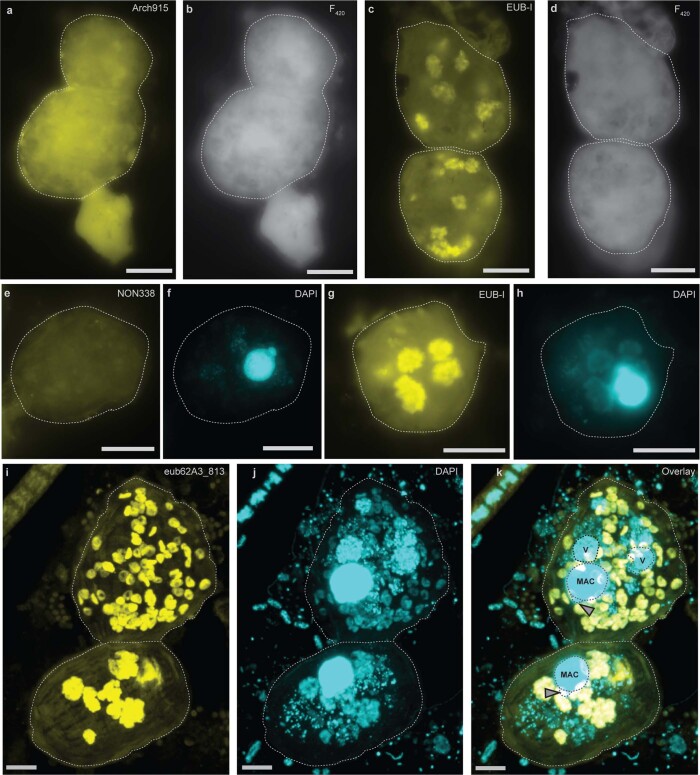
Fluorescence images of ciliates from Lake Zug. **a**–**e**, **g**, Epifluorescence images of ciliates from Lake Zug (2018, 180 m) after hybridization with a general probe for archaea (**a**) (Arch915), most bacteria (**c**, **g**) (EUB-I) and a negative control (**e**) (NON338). F_420_ autofluorescence (**b**, **d**) (in grey-scale) and Arch915 probe (**a**) images did not show discrete signals indicative of endosymbiotic methanogenic archaea. By contrast, clear signals of intracellular bacteria (endosymbiotic or in food vacuoles) are visible with the EUB-I probe (**c**, **g**). **f**, **h**, **j**, DAPI images show presence of macronucleus and micronucleus as well as endosymbionts. **i**–**k**, Confocal laser scanning microscopy images of two ciliates after hybridization with ‘*Ca*. Azoamicus’-specific oligonucleotide probe (eub62A3_813) (yellow) and counterstaining with DAPI (cyan). Overlay (**k**) and individual channels (eub62A3_813 (**i**) and DAPI (**j**)) from the same field of view; fluorescence images from a *z*-stack were projected into 2D for visualization. Two morphotypes of ‘*Ca*. A. ciliaticola’ are visible on the images: evenly distributed ovoid cells (1.5–2-μm long) and densely packed, irregular clusters (3–10-μm diameter) of cells. The macronucleus (MAC) with attached micronucleus (grey arrow) and putative food vacuoles (V) of the ciliate are outlined in the overlay image (**k**). In all images, ciliate cells are outlined with grey dotted lines (corresponding fields of view in **a** and **b**; **c** and **d**; **e** and **f**; **g** and **h**; and **i** and **k**). Scale bars, 5 μm.

**Extended Data Fig. 3:**
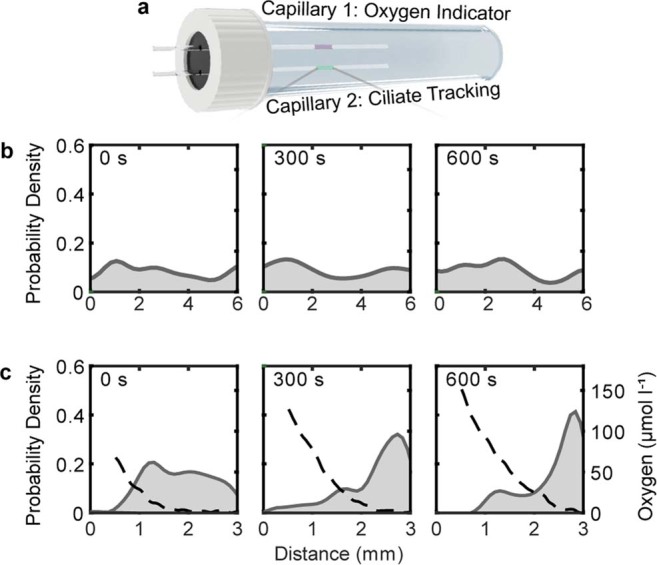
Negative aerotactic behaviour of ciliate hosts exposed to varying concentrations of oxygen. **a**, Schematic of a two-capillary experimental setup that allows for simultaneous oxygen measurements and ciliate tracking. **b**, Reference experiment showing the positioning of ciliates under anoxic conditions on the basis of 3 measurements after 0 s, 300 s and 600 s. The positioning is expressed as probability density and is defined as the percentage of the ciliates at a specific distance to the anoxic bubble. **c**, Chemotaxis experiments in which ciliates were exposed to a gradient of increasing oxygen concentration. These data were extracted from the video in Supplementary Video 2 with simultaneous ciliate tracking and oxygen concentration determination.

We observed a high abundance of motile unicellular eukaryotes throughout the anoxic layer (Fig. 1, Extended Data Fig. 1, Supplementary Video 1). Many of these eukaryotes contained macro- and micronuclei and had the typical morphology of ciliates (Fig. 1, Extended Data Fig. 2). Their abundances increased from about 6,000 cells per litre at the base of the oxycline (a depth of 160 m) to around 25,000 cells per litre within the core of the anoxic hypolimnion (a depth of about 180 m) (Supplementary Table 1). The ciliates exhibited negative aerotaxis, which indicates an obligate anaerobic lifestyle (Extended Data Fig. 3). In some cases, we observed fragmented and densely packed 4′,6-diamidino-2-phenylindole (DAPI) signals that might have depicted microbial prey in food vacuoles (Fig. 1, Extended Data Fig. 2). However, a substantial fraction of the ciliates observed in the anoxic hypolimnion also contained multiple intracellular DAPI signals that were reminiscent of endosymbionts. These putative endosymbionts did not show the typical cofactor F_420_ autofluorescence that is indicative of methanogen–hydrogenosome assemblages (which are often found in anaerobic ciliates) nor did they hybridize with a general archaeal oligonucleotide probe (Extended Data Fig. 2). We further confirmed the absence of methanogenic archaea in the ciliate by single-ciliate transcriptome sequencing (Supplementary Table 13, Supplementary Discussion). Instead, we obtained clear fluorescent signals after hybridization with a general bacteria-specific oligonucleotide probe (EUB-I) (Supplementary Table 3), which identified the potential ciliate endosymbionts as bacteria (Extended Data Fig. 2).

## Endosymbiont and host phylogeny

To identify the putative endosymbiont, we used DNA extracted from the anoxic lake water for metagenome sequencing (Supplementary Table 4). We identified a small (about 290 kb), circular metagenome-assembled genome with typical endosymbiotic features (as described in ‘Bacterial genome with endosymbiotic features’). The 16S rRNA gene sequence retrieved from the endosymbiont circular metagenome-assembled genome shared the highest identities (about 86%) with *Legionella clemsonensis* and ‘*Candidatus* Berkiella cookevillensis’ C99, which is an intranuclear bacterium of *Acanthamoeba polyphaga*. Given the high degree of sequence divergence between the endosymbiont and related bacteria, we named this organism ‘*Candidatus* Azoamicus ciliaticola’ (see Methods for etymology).

**Extended Data Fig. 4:**
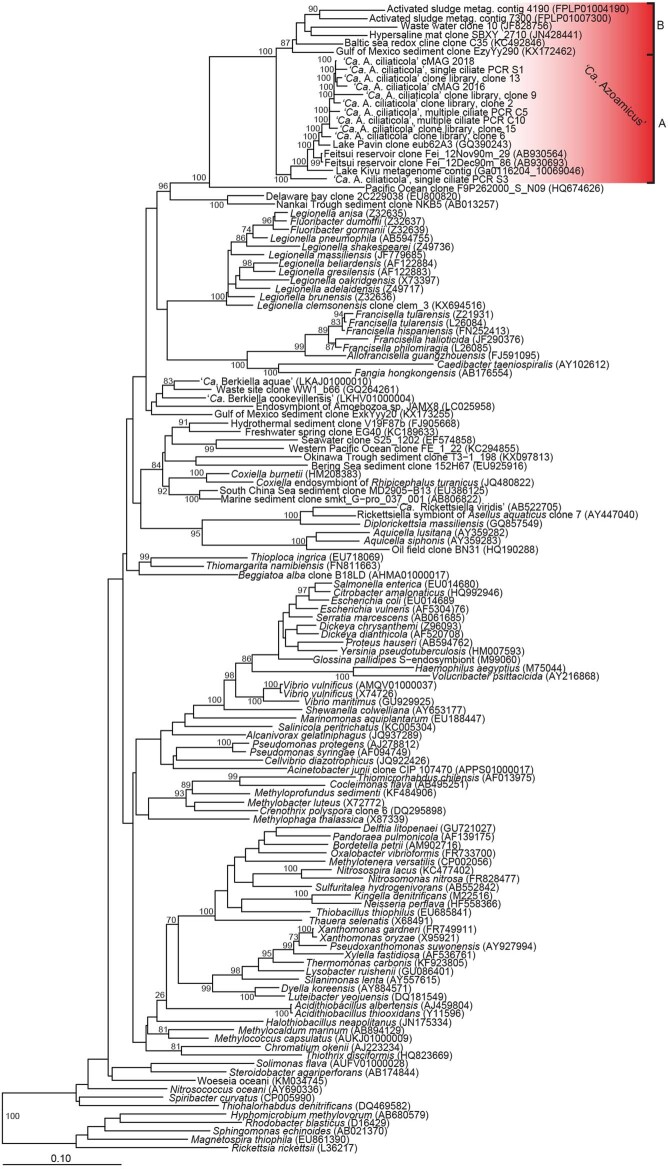
Phylogenetic affiliation of ‘*Ca*. A. ciliaticola’ within the Gammaproteobacteria. 16S rRNA gene sequence-based maximum-likelihood phylogenetic tree of ‘*Ca*. A. ciliaticola’ and other members of the Gammaproteobacteria. The circular metagenome-assembled genome-derived 16S rRNA gene sequences (cMAG 2016 and cMAG 2018) (details are provided in Supplementary Discussion), as well as ‘*Ca*. A. ciliaticola’ Sanger sequences retrieved from a clone library (clones 2, 6, 9, 13 and 15; details are provided in Methods), and amplified from individual (S1 and S3) or combined ciliates (C5 and C10), were included in the tree. ‘*Candidatus* Azoamicus’ subgroup A and B are highlighted in red. Four sequences belonging to the Alphaproteobacteria served as outgroup (*Rickettsia rickettsia*, L36217; *Magnetospira thiophila*, AB021370; *Hyphomicrobium methylovorum*, AB680579; and *Rhodobacter blasticus*, D16429). Bootstrap values >70% (out of 100 resamplings) are shown in front of the respective nodes, and the scale bar indicates nucleotide substitutions per site.

Our phylogenetic analysis showed that ‘*Ca*. A. ciliaticola’ belongs to the understudied eub62A3 group of the Gammaproteobacteria (Fig. 1e, Extended Data Fig. 4). The eub62A3 group (which we refer to as the ‘*Candidatus* Azoamicus’ group) probably represents its own order within Gammaproteobacteria. The ‘*Ca*. Azoamicus’ group appears to consist of at least two distinct subgroups. Sequences affiliated with subgroup A are closely related to ‘*Ca*. A. ciliaticola’ (98.5–99.1% sequence identity) and have been retrieved from geographically distant anoxic freshwater lakes and reservoirs (Fig. 1e). Subgroup B contains sequences that are more divergent from ‘*Ca*. A. ciliaticola’ (89.5–92.9% sequence identity) and that have been retrieved from more diverse aquatic habitats (Fig. 1e).

**Extended Data Fig. 5:**
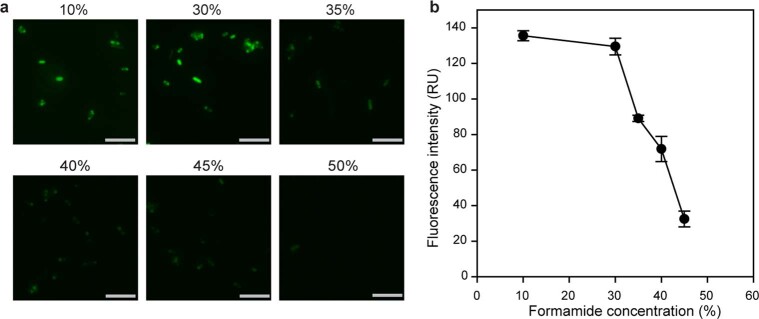
eub62A3_813 FISH probe optimization using clone-FISH. **a**, Fluorescence pictures of *E*. *coli* cells transcribing the 16S rRNA gene of ‘*Ca*. A. ciliaticola’ hybridized at different formamide concentrations (10%, 30%, 35%, 40%, 45% and 50%) with ‘*Ca*. A. ciliaticola’-specific probe eub62A3_813. Scale bar, 10 μm. **b**, Formamide melting curve for probe eub62A3_813. Centre values show average fluorescence intensity and error bars represent s.e. (10%, *n* = 28 cells; 30%, *n* = 22 cells; 35%, *n* = 26 cells; 40%, *n* = 10 cells; 45%, *n* = 4 cells). The fluorescence intensity of cells hybridized at 50% formamide could not be determined owing to the low intensity, and was therefore not included in the formamide melting curve. Source data

**Extended Data Fig. 6:**
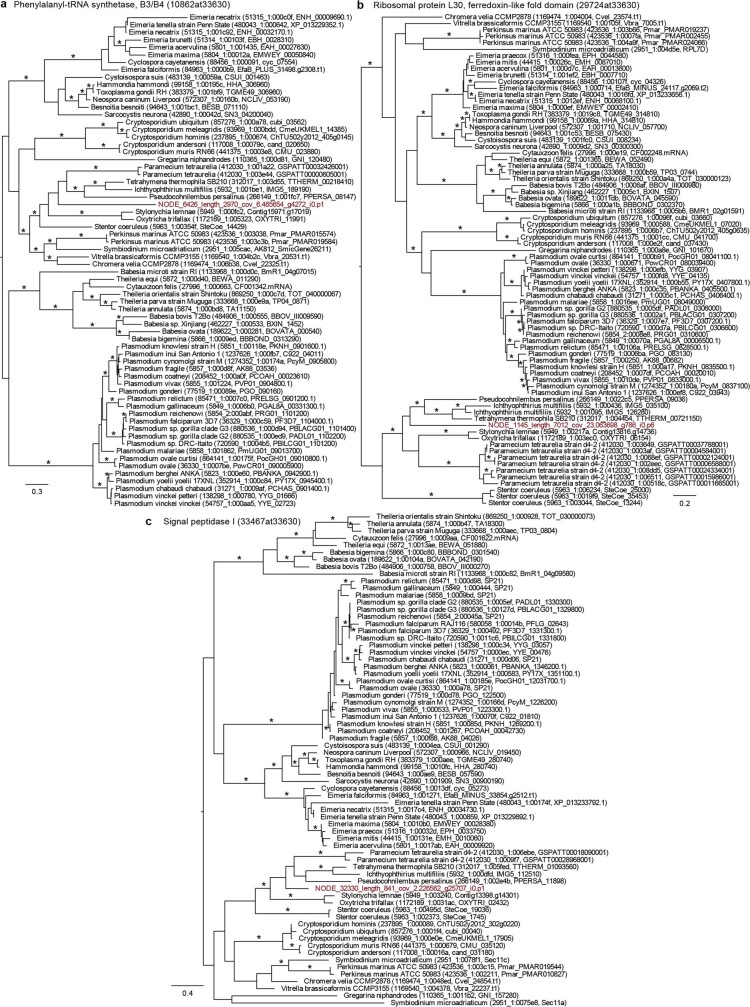
Ciliate host phylogeny based on three different benchmarking universal single-copy orthologues. **a**–**c**, The orthologue group name and identifier is shown at the top of each phylogenetic tree (10862at33630 (**a**), 29724at33630 (**b**) and 33467at33630 (**c**)). Comma-separated accessions in parentheses at tree leaves represent the ‘int_prot_id‘ and ‘pub_gene_id' as provided by orthoDB. The corresponding open reading frame representing the ciliate host is highlighted in red. Asterisks highlight branches with a Shimodaira–Hasegawa-like approximate likelihood ratio test value ≥ 80% and an ultrafast bootstrap support ≥ 95%. Phylogenies were rooted on the basis of the minimal ancestor deviation method. Scale bars indicate amino acid substitutions per site.

We used a fluorescently labelled ‘*Ca*. Azoamicus’-specific oligonucleotide probe (eub62A3_813) (Extended Data Fig. 5, Methods, Supplementary Table 3), and found that the endosymbionts were exclusively located inside small ovoid (25.4 ± 3.2-μm long, 19.5 ± 2.0-μm wide (±s.d. from average); *n* = 9 cells) ciliate hosts (Fig. 1, Extended Data Fig. 2). In 2018, all the ciliates we found at 180 m depth contained the ‘*Ca*. A. ciliaticola’ symbiont (Supplementary Table 1). We extracted DNA from individually picked ciliates from the deepest depth (189 m, which was sampled in 2019) and amplified ‘*Ca*. A. ciliaticola’ 16S rRNA gene using specific primers (Supplementary Table 2). We successfully retrieved sequences that were closely related to ‘*Ca*. A. ciliaticola’ (96.5–99.9% identity) from the picked ciliates. Our analysis of the partial 18S rRNA gene amplified from the same DNA extracts using ciliate-specific primers^28^ (Supplementary Fig. 1) identified all of the picked ciliates as members of the class Plagiopylea within the Ciliophora phylum (Fig. 1f). We further confirmed their Ciliophora affiliation by analyses of single-copy orthologous gene transcripts from single-ciliate transcriptomes (Extended Data Fig. 6). Plagiopyleans are anaerobic or micro-aerobic ciliates that are typically found in anoxic freshwater or marine habitats. They are generally thought to rely on hydrogenosomes, and some host methanogenic archaea^29^. The class Plagiopylea is phylogenetically divided into two clades^30^. One clade contains well-described members of the order Plagiopylida (such as *Plagiopyla frontata* and *Trimyema compressum*), and the second clade contains our ‘*Ca*. A. ciliaticola’-associated plagiopylean ciliate as well as diverse 18S rRNA gene sequences from freshwater and marine anoxic habitats (Fig. 1f). Our phylogenetic analyses thus indicated that ‘*Ca*. A. ciliaticola’ represents an endosymbiont of an anaerobic freshwater plagiopylean ciliate.

## Bacterial genome with endosymbiotic features

**Fig. 2:**
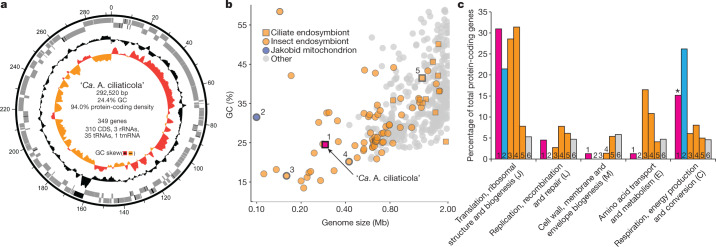
Features of the genome of ‘*Ca*. A. ciliaticola’. **a**, Circular genome plot of ‘*Ca*. A. ciliaticola’, showing (from inside to outside) GC skew (red, positive; light orange, negative), GC content, RNA (black) and protein-coding genes (grey) encoded on forward or reverse strand of the genome (in kb). **b**, Relationship between genome size and GC content of complete prokaryotic genomes deposited at GenBank (cut-off at 2 Mb; accessed May 2018). Genomes of ‘*Ca*. A. ciliaticola’, *Jakoba libera* mitochondrion, and endosymbionts of insects and ciliates are highlighted. 1, ‘*Ca*. A. ciliaticola’; 2, *J. libera* mitochondrion; 3, ‘*Ca*. Carsonella ruddii’ PV; 4, *Buchnera aphidicola*; 5, *Caedibacter taeniospiralis*. *Legionella clemsonensis* is not shown in this panel as its genome size (3.27 Mb) exceeds the depicted range. **c**, Comparison of the number of genes in selected functional gene categories (as a percentage of total protein-coding genes) (Supplementary Table 9) between ‘*Ca*. A. ciliaticola’ (magenta), *J. libera* mitochondrion (blue), selected insect and ciliate endosymbionts (orange), and *Legionella clemsonensis* (grey) as an example of a related free-living bacterium. Numbers are as described in **b**; 6 denotes *L. clemsonensis*. The asterisk highlights the presence of an anaerobic respiratory chain. Single-letter codes of COG functional categories are shown in parentheses. Source data

The closed genome of ‘*Ca*. A. ciliaticola’ is very small (292,520 bp), and has a notably low average G + C content (24.4%) and a high protein-coding density (94.0%) (Fig. 2a). It has retained 310 protein-coding genes, 1 ribosomal RNA operon (16S, 23S and 5S), 35 transfer RNAs that decode all 20 standard amino acids (genetic code 11) and 1 transfer-messenger RNA; as such, it is, to our knowledge, the smallest protist endosymbiont genome reported to date. Genomes of a similar size and with similar general features have thus far been found only in obligate endosymbionts of insects^31,32^ (Fig. 2b). On the basis of these similarities, we concluded that ‘*Ca*. A. ciliaticola’ is an obligate endosymbiont.

**Extended Data Fig. 7:**
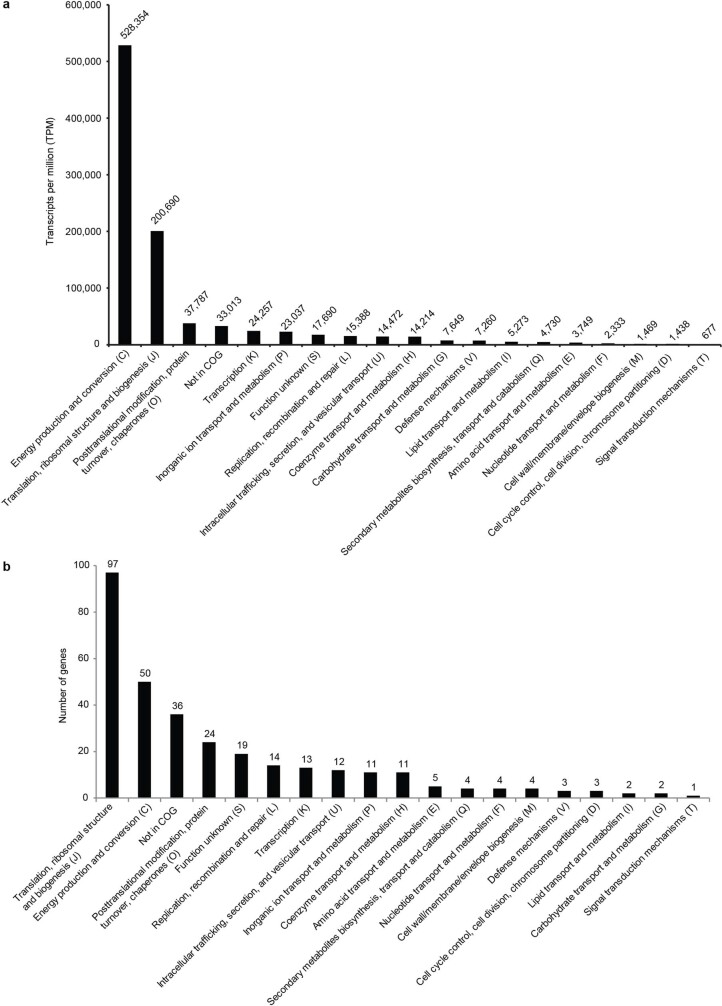
Total gene transcription and number of genes per functional category. **a**, Bar chart showing the sum of TPM (indicated above each bar) of ‘*Ca*. A. ciliaticola’ genes within different functional categories. For the TPM calculation, the Lake Zug bulk water metatranscriptome data (MT_18_C) were used. Genes with several assigned categories were not included in the analysis. **b**, Bar chart showing the number of genes encoded in the genome of ‘*Ca*. A. ciliaticola’ assigned to different functional categories on the basis of clusters of orthologous genes analysis. The number of genes within each functional category is indicated above each bar. Source data

‘*Candidatus* A. ciliaticola’ encodes a similar proportion of genes related to translation, replication and repair to that encoded by other highly reduced endosymbionts of ciliates and insects (Fig. 2c). The genome encodes several subunits of the DNA polymerase III holoenzyme (*dnaE*, *dnaQ* and *dnaX*), DNA-directed RNA polymerase (*rpoA*, *rpoB*, *rpoC*, *rpoD*, *rpoH* and *rpoZ*) and a complete gene set of 30S and 50S ribosomal proteins. These genes were highly transcribed and accounted for around 20% of cumulative transcripts per million (Extended Data Fig. 7). Therefore, ‘*Ca*. A. ciliaticola’—despite its reduced genome—appears to be capable of self-replication inside its host.

By contrast, the ‘*Ca*. A. ciliaticola’ genome exhibits extensive loss of the genes that underlie cell wall, membrane and envelope biogenesis. The genetic repertoire for the biosynthesis of vitamins and amino acids is also markedly reduced, which is notably different from other endosymbionts of insects or ciliates (Fig. 2c, Extended Data Fig. 7). As many endosymbionts provide essential amino acids or vitamins to supplement the nutritionally poor diets of their hosts, they often retain extensive biosynthetic pathways for these compounds^32^. The ‘*Ca*. A. ciliaticola’ genome encodes only three copies of a tyrosine and tryptophan transporter (*tyrP*) and a glutamate and γ-aminobutyrate (glutamate/γ-aminobutyrate) antiporter that might serve to import aromatic amino acids and glutamate into the cell. Notably, ‘*Ca*. A. ciliaticola’ appears to depend on its host for most of the other essential cellular building blocks (for example, nucleotides, phospholipids and lipopolysaccharides), as no genes for their biosynthesis are encoded in the genome. It is therefore puzzling that, similar to other obligate endosymbionts^33^, the ‘*Ca*. A. ciliaticola’ genome largely lacks transport systems for these metabolites (Supplementary Tables 5, 6, Supplementary Discussion). Other genes that have previously been shown to be related to nutritional (for example, those that encode pectinases^34^) or defensive symbioses known from ciliates^35^ are also absent from the genome.

## Anaerobic respiration and energy metabolism

**Fig. 3:**
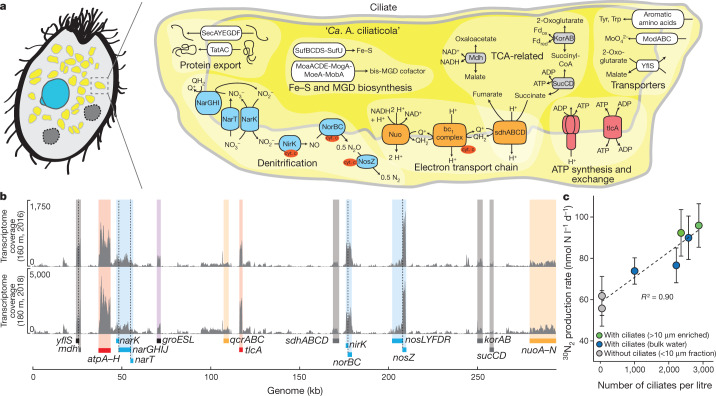
Metabolic potential and activity of ‘*Ca*. A. ciliaticola’. **a**, Genome-inferred metabolic potential related to anaerobic respiration via denitrification, ATP generation and exchange, tricarboxylic acid (TCA) cycle, iron–sulfur-cluster (Fe–S) and bis-molybdopterin guanine dinucleotide (bis-MGD) biosynthesis, protein export and other transporters. The presence of a double membrane is presumed by analogy with mitochondria. **b**, Metatranscriptome coverage of two metatranscriptomic datasets, from samples obtained in 2016 and 2018, of the ‘*Ca*. A. ciliaticola’ genome. There is a highly consistent transcriptome profile between years and an exceptionally high transcription of genes related to energy conservation, ATP production and transport, chaperones (*groE*, *groS* and *groL*) and denitrification (orange, red, purple and blue shading, respectively). Coverage was corrected for sequencing depth; the higher coverage in the 2018 sample is due to the higher endosymbiont abundance in that year. Genes related to denitrification (blue), electron transport chain (orange), tricarboxylic acid cycle (grey) and ATP generation as well as transport (red) are highlighted. The highlighted genes account for more than 55% of all transcripts. Further information is provided in Supplementary Table 5). **c**, Correlation of ciliate abundance and denitrification rates (^30^N_2_ production) in water from the anoxic hypolimnion (189 m, May 2019) with and without ciliates. Each data point represents a volumetric denitrification rate calculated from the linear regression of six individual time points of an experiment. Error bars represent s.e. from linear regression. Denitrification rates in incubations without ciliates are due to the activity of denitrifying bacteria that are still present in the water. Source data

In contrast to most other functional gene categories, genes related to respiration and energy production and conversion (Figs. 2c, 3a, Supplementary Table 5) are abundantly retained in the ‘*Ca*. A. ciliaticola’ genome. ‘*Candidatus* A. ciliaticola’ encodes a complete ATP-generating electron transport chain, including NADH dehydrogenase (*nuoA* to *nuoN*), cytochrome *bc*_1_ complex (*qcrA*, *qcrB* and *qcrC*) and ATP synthase (*atpA* to *atpH*). In many endosymbionts, genes related to respiration and energy production and conversion are affected by marked losses; some endosymbionts are even thought to be ATP parasites^32^. Instead, a substantial fraction of the ‘*Ca*. A. ciliaticola’ genome is dedicated to energy production and conversion, which is a feature that it shares with all known mitochondrial genomes (Fig. 2c).

The ‘*Ca*. A. ciliaticola’ genome does not encode genes for aerobic respiration, such as terminal oxidases (for example, cytochrome *c* oxidase and cytochrome *bd* oxidase). In principle, the terminal oxidase genes could have been relocated to the nuclear genome (as is the case with many endosymbiont genes). However, electron transport chain complexes I, II, IV and V are conserved in the mitochondrial core genome across many eukaryotes, and it is expected that genes that encode components of the electron transport chain are amongst the least likely to be transferred to the nucleus^11^. We also did not find transcripts for mitochondrially encoded cytochrome *c* oxidase in the host transcriptome (Supplementary Table 10, Supplementary Discussion). This confirms the obligate anaerobic nature of the host, as was already indicated by its negative aerotactic behaviour (Extended Data Fig. 3).

The ‘*Ca*. A. ciliaticola’ genome encodes a complete gene set for respiratory denitrification (Fig. 3a, Supplementary Table 5). The gene set contains all four core enzymes: nitrate reductase (*narG*, *narH* and *narI*), copper-containing nitrite reductase (*nirK*), nitric oxide reductase (*norB* and *norC*) and nitrous oxide reductase (*nosZ*). These genes are complemented by a wide variety of accessory proteins, such as cytochromes *c*_4_ and *c*_5_ for redox coupling, enzyme maturation factors (*narJ*, *nosR*, *nosD*, *nosF*, *nosY* and *nosL*), and nitrate and nitrite transporters (*narK* and *narT*). ‘*Candidatus* A. ciliaticola’ encodes a molybdate import system (*modA*, *modB* and *modC*) and a pathway for the biosynthesis of bis-molybdopterin guanine dinucleotide, which is an essential cofactor of nitrate reductase. In eukaryotes, this pathway is—for the most part—a cytosolic process that is encoded by nuclear genes^36^. Similarly, a pathway of iron–sulfur-cluster biosynthesis (Suf-type) (*sufB*, *sufC*, *sufD* and *sufS*) is also encoded in the ‘*Ca*. A. ciliaticola’ genome; this was the only pathway that we detected for iron–sulfur-cluster biosynthesis in the ciliate host. We did not detect transcripts that pertain to the Isc-type pathway of iron–sulfur-cluster biosynthesis (which operates in hydrogenosomes, mitochondria and mitochondria-related organelles) in the single-ciliate transcriptome (Supplementary Table 11).

**Extended Data Fig. 8:**
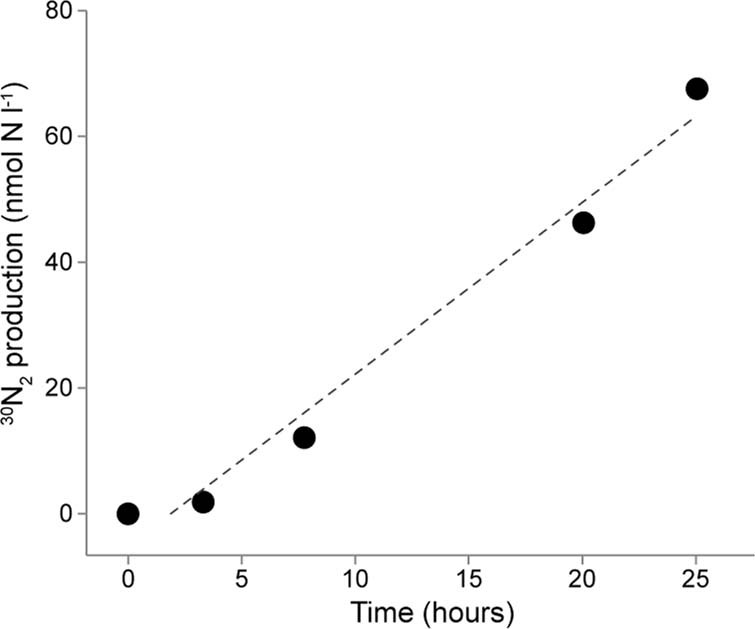
Linear production of ^30^N_2_ from ^15^N-labelled nitrate and nitrite. Incubation of Lake Zug water containing ciliates (sampled in May 2019 from 189 m) (Methods) amended with a mixture of ^15^N-labelled nitrate and nitrite. The production of ^30^N_2_ was followed over the course of 25 h. Source data

Although the trait of respiratory denitrification is widespread among free-living prokaryotes^37,38^, to our knowledge no obligate endosymbiont described to date encodes a denitrification pathway. We confirmed active denitrification by ciliates using ^15^N stable isotope incubations (Fig. 3c, Extended Data Fig. 8, Supplementary Discussion). Possible electron donors for denitrification might be di- and/or tri-carboxylates provided by the tricarboxylic acid cycle, such as succinate and malate (Supplementary Discussion). Malate is used by many known energy-producing anaerobic mitochondria or hydrogenosomes of ciliates or other protists^4^. The high transcription of malate dehydrogenase (*mdh*) and a putative 2-oxoglutarate and malate (2-oxoglutarate/malate) transporter (*yflS*) (Fig. 3b) indicates that malate also has a key role in the metabolism of ‘*Ca*. A. ciliaticola’. Similarly, genes related to energy conservation—including ATP synthase (*atpA* to *atpH*) and denitrification genes (*narG*, *narH*, *narI*, *nirK*, *norB*, *norC* and *nosZ*)—were among those showing the highest transcription (Fig. 3b) and, altogether, we found that more than half of the transcriptional effort of ‘*Ca*. A. ciliaticola’ was devoted to energy production and conversion (Extended Data Fig. 7).

**Extended Data Fig. 9:**
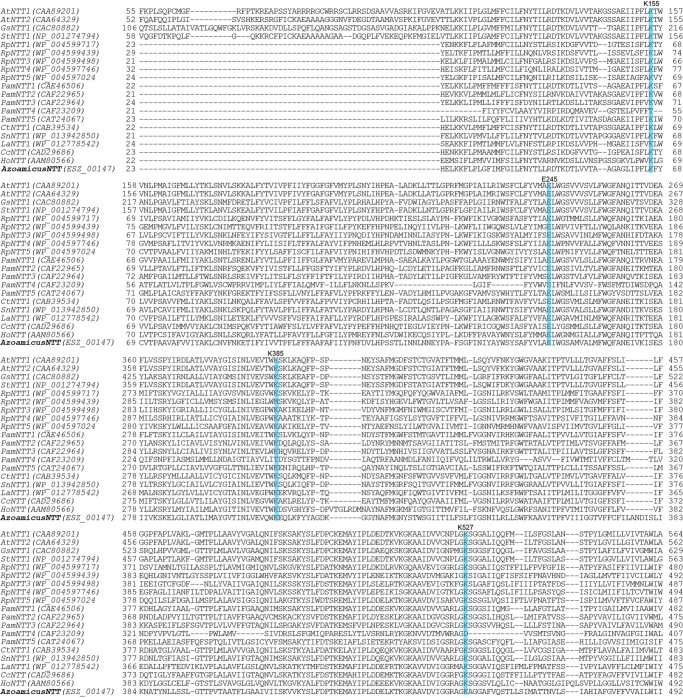
Multiple sequence alignment of ‘*Ca*. A. ciliaticola’ nucleotide transport protein with other bacterial and plastidic nucleotide transport proteins. Conserved residues that are important for transport efficiency, substrate specificity and counter exchange properties of ATP/ADP translocase from *Arabidopsis thaliana*^86^ are highlighted in blue, and numbered according to their position in *Arabidopsis* NTT1 (AtNTT1). All of these residues are conserved in the nucleotide transporter of ‘*Ca*. A. ciliaticola’. Accession codes are provided in parentheses, and further information regarding the aligned sequences is listed in Supplementary Table 8.

Crucially, the ‘*Ca*. A. ciliaticola’ genome also contains a putative ATP and ADP (hereafter, ATP/ADP) translocase gene (*tlcA*) that encodes a single-domain nucleotide transport protein. These nucleotide transport proteins have canonical roles in energy transfer in plastids and intracellular parasites, although these might not be their only functions^39^. The ‘*Ca*. A. ciliaticola’ nucleotide transport protein has several conserved residues that are critical for function and substrate specificity of ATP/ADP translocases (Extended Data Fig. 9, Supplementary Discussion). Moreover, the *tlcA* gene was among the ten highest transcribed ‘*Ca*. A. ciliaticola’ genes, together with other genes that are involved in energy conservation (Fig. 3b). On this basis, we speculate that the TlcA nucleotide transporter serves to exchange ATP and ADP between ‘*Ca*. A. ciliaticola’ and its host. As ‘*Ca*. A. ciliaticola’ lacks the ability to synthesize any nucleotides de novo, this nucleotide transport protein might also be involved in the uptake of nucleotides other than ATP and ADP.

As is known from other obligate endosymbionts, the genome of ‘*Ca*. A. ciliaticola’ has presumably preserved traits that are beneficial to the host. Given the fact that the highly reduced genome of ‘*Ca*. A. ciliaticola’ has retained an extensive genetic potential for energy metabolism, the main function of this endosymbiont thus appears to be the production of ATP for its host. In this respect, the function of ‘*Ca*. A. ciliaticola’ is strongly evocative of mitochondria and hydrogenosomes (that is, eukaryotic energy-providing organelles). On the basis of our combined metagenomic and transcriptomic results, we propose that ‘*Ca*. A. ciliaticola’ functions as a respiratory endosymbiont that conserves and provides energy from anaerobic respiration for its plagiopylean host.

## Evolution of the respiratory symbiosis

**Fig. 4:**
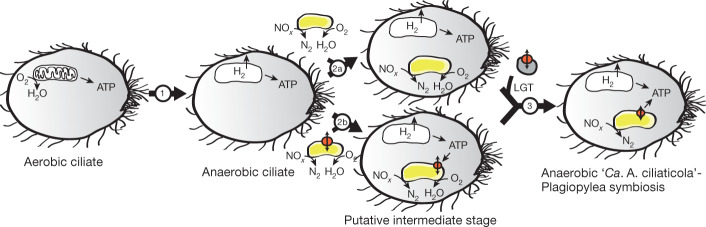
Hypothesized evolution of ‘*Ca*. A. ciliaticola’–ciliate symbiosis. (1) Secondary adaptation of an aerobic ciliate to an anoxic, nitrate-containing environment involved the deterioration of its aerobic mitochondria and the formation of hydrogenosomes. (2) Acquisition of a facultatively anaerobic, denitrifying bacterium that entered the anaerobic ciliate host either via endocytosis (2a) or as a parasite (2b). In the latter case, the ‘*Ca*. A. ciliaticola’ ancestor might have already possessed an ATP/ADP translocase for energy parasitism. (3) Alternatively, the ATP/ADP translocase was acquired at a later point via lateral gene transfer (LGT) from (for example) an alphaproteobacterial cosymbiont in the host cell. Persistent anoxic and nitrate-rich conditions would result in the loss of genes for oxygen respiration that gave rise to the present-day ‘*Ca*. A. ciliaticola’–Plagiopylea symbiosis. The presence of hydrogenosomes in the extant host is based on indications from single-ciliate transcriptome sequencing, and is yet to be conclusively confirmed.

Unicellular eukaryotes that have secondarily adapted to an anaerobic lifestyle often possess mitochondria-related organelles such as hydrogenosomes. These organelles have retained their energy-generating function—although they have often lost their genomes, and their enzymes are predominantly nuclear-encoded and expressed by the eukaryotic host^40^. When analysing the single-cell transcriptomes of the ciliate host, we found transcripts that potentially encode hydrogenosomal proteins (Supplementary Tables 11, 12, Supplementary Methods, Supplementary Discussion). This suggests that our ciliate host—as with all eukaryotes known to date—may contain remnants of a mitochondrial organelle, which is probably capable of hydrogenosomal metabolism (Fig. 4).

The putative presence of hydrogenosomes in the extant host implies that, at some point, its ancestor possessed aerobic mitochondria. However, in a host that is capable of aerobic respiration, the acquisition of a denitrifying endosymbiont is unlikely to have increased the fitness of the host sufficiently to warrant stable integration. It is thus more likely that the ciliate host was already a hydrogenosome-containing obligate anaerobe before the acquisition of the predecessor of ‘*Ca*. A. ciliaticola’ (Fig. 4). A fermenting eukaryote that possessed only rudimentary means of energy generation would have profited considerably from the acquisition of a bacterium with the capacity for anaerobic nitrate respiration and chemiosmotic ATP production. The positive effect of the ‘*Ca*. A. ciliaticola’ endosymbiont on the fitness of the ciliate host could explain our observation that only ‘*Ca*. A. ciliaticola’-containing ciliates were observed in the deep anoxic nitrate-containing waters of Lake Zug (Supplementary Table 1).

**Extended Data Fig. 10:**
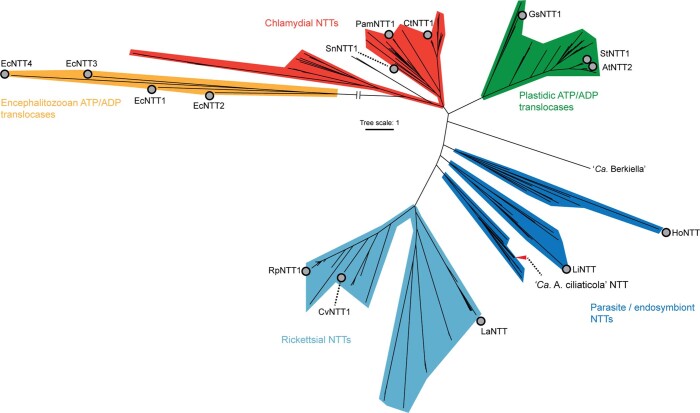
Phylogenetic affiliation of ‘*Ca*. A. ciliaticola’ ATP and ADP translocase and related nucleotide transporters. Maximum-likelihood phylogenetic tree based on amino acid sequences of bacterial and plastidic ATP/ADP translocases and related nucleotide transporters. ‘*Ca*. A. ciliaticola’ ATP/ADP translocase (red arrow) clusters with environmental sequences related to the Holosporales and *Caedibacter*. The colours of groups reflect the predominant phylogenetic affiliation of taxa within a group. Sequences that have been shown to translocate ATP and ADP are marked with grey dots (Supplementary Table 7). Pam, *Protochlamydia amoebophila*; Ct, *Chlamydia trachomatis*; Sn, *Simkania negevensis*; Li, *Lawsonia intracellularis*; Rp, *Rickettsia prowazekii*; La, *Liberibacter asiaticus*; Ec, *Encephalitozoon cuniculi*; Cv, *Caedimonas varicaedens*; Ho, *Holospora obtusa*; St, *Solanum tuberosum*; Gs, *Galdieria sulphuraria*; At, *Arabidopsis thaliana*. ATP/ADP translocases of *E. cuniculi* served as outgroup and are shown with truncated branch length, which is indicated as a break. Source data

The predecessor of ‘*Ca*. A. ciliaticola’ may have entered its host as a parasite, as many members of the related Legionellales and Francisellales groups are parasitic. Alternatively, the bacterial predecessor could have been acquired through endocytosis and then persisted intracellularly after managing to escape digestion, as has previously been suggested for some bacteria^41^. In either case, the protein that was probably fundamental for the integration of the bacterium into its host is the ATP/ADP transporter. Phylogenetic analyses showed that the putative ATP/ADP translocase of ‘*Ca*. A. ciliaticola’ clusters with mostly alphaproteobacterial nucleotide transporter sequences from uncharacterized metagenomic sequences related to Holosporales or *Caedibacter* (Extended Data Fig. 10), which are known to be intracellular parasites or endosymbionts of ciliates^42,43^. Considering that the free-living predecessor of ‘*Ca*. A. ciliaticola’ was a gammaproteobacterium, we speculate that the ATP/ADP translocase might have been obtained via lateral gene transfer from an alphaproteobacterial donor (for example, *Holospora*-related) (Supplementary Discussion). At this point, it is not clear whether the lateral gene transfer happened before or after the engulfment of the predecessor of ‘*Ca*. A. ciliaticola’ by the ciliate (Fig. 4).

In its extant form, ‘*Ca*. A. ciliaticola’ encodes only the denitrification respiratory chain. This is noteworthy as almost all pro- and eukaryotic denitrifying organisms known to date possess a hybrid respiratory chain that is also capable of oxygen respiration^38,44^. On this basis, it seems likely that the predecessor of ‘*Ca*. A. ciliaticola’ acquired by the ciliate host was a facultatively anaerobic denitrifying bacterium (Fig. 4). Subsequently, it might have lost its capacity for oxygen respiration when it became superfluous in a ciliate that exclusively inhabited anoxic waters. However, any gene loss in ‘*Ca*. A. ciliaticola’ might also be the result of genetic drift, which is known to affect obligate endosymbionts with small population sizes and restricted gene flow^31^.

Over the course of its existence, ‘*Ca*. A. ciliaticola’ has evolved into an obligate endosymbiont with a distinct role in respiration and energy generation for its host; in eukaryotes, these roles are traditionally reserved for mitochondria and related organelles. Even though the ciliate host might contain mitochondria-related organelles (that is, putative hydrogenosomes), it appears that quintessential mitochondrial functions related to respiration, the electron transport chain, oxidative phosphorylation and iron–sulfur-cluster biosynthesis are taking place in its endosymbiont.

‘*Candidatus* A. ciliaticola’ therefore represents an example of an obligate endosymbiont that has retained cellular functions that are markedly similar to those of mitochondria, although it did not originate from the mitochondrial line of descent. This discovery provokes the question of whether other prokaryotic endosymbionts may enable eukaryotes to tap into the broad pool of electron acceptors that is available in nature.

## Methods

No statistical methods were used to predetermine sample size. The experiments were not randomized, and investigators were not blinded to allocation during experiments and outcome assessment.

### Etymology

The designation ‘Azoamicus’ combines the prefix *azo*- (New Latin, pertaining to nitrogen) with *amicus* (Latin, masculine noun, friend); thus giving *azoamicus* (‘friend that pertains to nitrogen’), alluding to its role as denitrifying endosymbiont. ‘Ciliaticola’ combines ciliate (referring to a group of ciliated protozoa) with the suffix -*cola* (derived from the Latin masculine noun *incola*, dweller, inhabitant), thus meaning ‘dwelling within a ciliate’.

### Geochemical profiling

We carried out sampling for geochemical profiling in September 2016 and October 2018 at a single station located in the deep, southern lake basin of Lake Zug (about 197-m water depth) (47° 06′ 00.8′′ N, 8° 29′ 35.0′′ E). In September 2016, we used a multi-parameter probe to measure conductivity, turbidity, depth (pressure), temperature and pH (XRX 620, RBR). Dissolved oxygen was monitored online with normal and trace micro-optodes (types PSt1 and TOS7, Presens) with detection limits of 125 and 20 nM, respectively, and a response time of 7 s. In October 2018, we used a CTD (CTD60, Sea&Sun Technology) equipped with a Clark-type oxygen sensor (accuracy ± 3%, resolution 0.1%) to record oxygen profiles.

### Sample collection

Water for bulk DNA and RNA analyses was collected in September 2016 and October 2018. Sample collection for DNA and RNA extraction in September 2016 has previously been described^46^. In October 2018, water was sampled with a Niskin bottle (Hydro-Bios) from 160 m, 170 m and 180 m. For each depth, 2 l of lake water was directly filtered on board our boat onto 0.22-μm Sterivex filter cartridges (Merck Millipore) using a peristaltic pump, subsequently purged with RNAlater preservation solution (Life Technologies) and stored at −20 °C until further processing. For fluorescence in situ hybridization (FISH) analyses, water from the same depths was fixed on board the boat with formaldehyde (1.5% final concentration; Electron Microscopy Sciences) and incubated in a chilled cool box for about 6 h before filtration onto 3-μm polycarbonate filters (Merck Millipore). Additional FISH samples using the same approach were collected in May 2019 from 189 m water depth.

Water for incubation experiments and single-ciliate PCR was sampled in May 2019 from 189-m water depth using a 10 l Go-Flo bottle (General Oceanics), filled into 2.5-l glass bottles without headspace, closed with butyl rubber stoppers and kept cold (at about 4 °C) and dark until further handling. During sampling, oxygen contamination was minimized by overflowing the bottle with anoxic lake water.

For combined FISH and differential interference contrast microscopy analyses, individual live ciliates were picked from lake water (from 186-m depth, collected February 2020) and directly fixed on microscope slides. In brief, microscope slides were treated with 0.1 mg ml^−1^ poly-l-lysine for 10 min at room temperature, washed with MilliQ water and dried. Ciliates were pre-enriched by gravity flow of bulk lake water through a 5-μm membrane filter and picked using a glass capillary under a binocular microscope. Picked ciliates were transferred into a droplet of formaldehyde (2% in 0.1× PBS, pH 7.6) on poly-l-lysine-coated microscope slides, incubated (for 1 h at room temperature) and washed with MilliQ water. FISH was performed as described in ‘Double-labelled oligonucleotide probe fluorescence in situ hybridization and microscopy’.

### Nutrient measurements

Water samples for measurements of nutrients (ammonium, NO_*x*_ and nitrite) were retrieved with a syringe sampler from 15 discrete depths at and below the base of the oxycline. Forty ml of water was directly injected into a 50-ml Falcon tube containing 10 ml of OPA reagent for fluorometric ammonium quantification^47^. In 2018, ammonium concentration was determined using the same method, except that the lake water was immediately sterile-filtered after sampling and frozen at −20 °C until further processing. For NO_*x*_ quantification, 10 ml of water was sterile-filtered into a 15-ml Falcon tube and combined nitrate and nitrite concentration was determined by a commercial QuAAtro Segmented Flow Analyzer (SEAL Analytical).

### Clone library construction and Sanger sequencing

‘*Candidatus* A. ciliaticola’-specific 16S rRNA gene primers were designed on the basis of the ‘*Ca*. A. ciliaticola’ circular metagenome-assembled genome sequence. Primers targeted the intergenic spacer regions about 50 bp up- and downstream of the 16S rRNA gene, resulting in a 1,568-bp-long PCR product. For clone library construction, the ‘*Ca*. A. ciliaticola’ 16S rRNA gene was amplified by a nested PCR approach from the same DNA extract used for metagenome sequencing obtained in September 2016 from 160-m water depth using the newly designed ‘*Ca*. A. ciliaticola’-specific primers (eub62A3_29F and eub62A3_1547R) followed by PCR amplification with general bacterial 16S rRNA gene primers (8F and 1492R) (Supplementary Table 2). Cloning and construction of the clone library is described in more detail in Supplementary Methods. Inserts of purified plasmids from five clones were sequenced by Sanger sequencing using the BigDye Terminator v.3.1 sequencing kit (Thermo Fisher Scientific) and primers M13f or M13r. The sequencing PCR contained 3 μl purified plasmid, 0.5 μl 10× sequencing buffer, 0.5 μl primer (10 μM) and 1 μl BigDye reagent. The PCR reactions were performed as follows: 99 cycles (1 °C s^−1^ ramp) of denaturation (10 s at 96 °C), annealing (5 s at 60 °C) and elongation (4 min at 60 °C). The PCR products were purified using gel filtration (Sephadex G-50 Superfine, Amersham Bioscience) followed by Sanger sequencing (3130xl genetic analyser, Applied Biosystems). The Sanger sequences were quality-trimmed and assembled using Sequencher v.5.4.6 and standard settings before trimming vector and primer sequences.

### Probe design for fluorescence in situ hybridization

To visualize ‘*Ca*. A. ciliaticola’ cells in the environment, we designed a specific FISH probe on the basis of the ‘*Ca*. A. ciliaticola’ circular metagenome-assembled genome 16S rRNA gene sequence and closely related sequences within the clade eub62A3. The ‘*Ca*. A. ciliaticola’ 16S rRNA gene sequence was imported into Arb^48^ v.6.1 and aligned to the SILVA SSU Ref NR 99 132 database using the SINA-Aligner^49^. A FISH probe specific for ‘*Ca*. A. ciliaticola’ and most members of clade eub62A3 was designed (probe eub62A3_813 5′ CTAACAGCAAGTTTTCATCGTTTA 3′) (Supplementary Table 3) using the probe design tool implemented in Arb, and further manually refined and evaluated in silico using MathFISH^50^. The newly designed probe eub62A3_813 targets ‘*Ca*. A. ciliaticola’ and 78% of the ‘*Ca*. Azoamicus’ subgroup A and B sequences included in SILVA SSU Ref NR 99 138 (7 out of 9; the 2 sequences that are not targeted belong to ‘*Ca*. Azoamicus’ subgroup B), and shows no nontarget hits. Some sequences in the database had only 1 or 2 weak mismatches (<0.5 weighted mismatches) to the probe sequence. To ensure specificity in our FISH experiments and exclude the detection of nontarget organisms, we designed unlabelled competitor probes for these sequences with weak mismatches to the probe (competitor 1, 5′ CTAACAGCAAGTTCTCATCGTTTA 3′ and competitor 2, 5′ CCAACAGCAAGTTCTCATCGTTTA 3′) (Supplementary Table 3). These competitor probes were included in all FISH experiments (excluding clone-FISH). To further ensure that no nontarget organisms were present in our samples, we searched for 16S rRNA gene reads with perfect matches against the eub62A3_813 probe sequence in one metagenome (Zug 2018, 180 m). Almost all of the reads (82 out of 86) had >98% identity with the 16S rRNA gene sequence of ‘*Ca*. A. ciliaticola’. The remaining reads shared >94% identity with ‘*Ca*. A. ciliaticola’ and all shared as top hits sequences belonging to ‘*Ca*. Azoamicus’ subgroup A when blasted against the NCBI nr database (accessed June 2020).

### Clone-FISH

Clone-FISH was performed as previously described^51^. In brief, a purified plasmid containing the ‘*Ca*. A. ciliaticola’ 16S rRNA gene sequence (as described in ‘Clone library construction and Sanger sequencing’) in the correct orientation (confirmed by PCR using M13F and 1492R primers) was transformed into electrocompetent *Escherichia coli* JM109(DE3) cells (Promega) by electroporation using the Cell Porator and Voltage Booster System (Gibco) with settings 350 V, 330 μF capacitance, low ohm impedance, fast charge rate and 4 kΩ resistance (Voltage Booster). After electroporation, cells were transferred into SOC medium (Sigma Aldrich), incubated for 1 h at 37 °C and plated onto LB plates containing 100 mg l^−1^ kanamycin. After incubation overnight at 37 °C, 4 clones were picked and the presence of the insert was checked with PCR (primers M13F and 1492R) as described in ‘Clone library construction and Sanger sequencing’, followed by gel electrophoresis. An insert-positive clone was selected at random and grown in LB medium containing 100 mg l^−1^ kanamycin at 37 °C until optical density at 600 nm reached 0.37. Transcription of the plasmid insert was induced using isopropyl β-d-1-thiogalactopyranoside (IPTG) (1 mM final concentration). After addition of IPTG, cells were incubated for 1 h at 37 °C followed by addition of 170 mg l^−1^ chloramphenicol and subsequent incubation for 4 h. Cells were collected by centrifugation, fixed in 2% formaldehyde solution for 1 h at room temperature, washed and stored at 4 °C in phosphate-buffered saline (pH 7.4) containing 50% ethanol until further processing. Formamide melting curves^52^ were carried out using a ‘*Ca*. Azoamicus’-specific, HRP-labelled probe (eub62A3_813) (Extended Data Fig. 5). In brief, cells were applied to glass slides. Permeabilization with lysozyme, peroxidase inactivation, hybridization (10%, 30%, 35%, 40%, 45% and 50% formamide) and tyramide signal amplification (Oregon Green 488) was performed as previously described^53^. For each formamide concentration, images of three fields of view were recorded using the same exposure time for all formamide concentrations, which was optimized at 10% formamide all same settings using an Axio Imager 2 microscope (Zeiss) and analysed using Daime 2.1^54^.

### Double-labelled oligonucleotide probe fluorescence in situ hybridization and microscopy

Hybridization with double-labelled oligonucleotide probes (terminally double-labelled with Atto488 dye; details of the probe are in Supplementary Table 3) (Biomers) and counterstaining with DAPI was performed as previously described^55^. In brief, samples (either cut filter sections or ciliates picked and fixed on a glass slide, as described in ‘Sample collection’) were incubated in hybridization buffer containing 25% formamide and 5 ng DNA μl^−1^ probe (the same concentration was used for eub62A3_813 competitor 1 and 2) for 3 h at 46 °C, and subsequently washed in prewarmed washing buffer (5 mM EDTA, 159 mM NaCl) for 30 min at 48 °C. After a brief MilliQ water wash, samples were incubated for 5 min at room temperature in DAPI solution (1 μg ml^−1^), briefly washed in MilliQ water and air-dried. Filter sections were mounted onto glass slides. Samples were embedded in Prolong Gold Antifade Mountant (Thermo Fisher Scientific), and left to cure for 24 h. Confocal laser scanning and differential interference contrast microscopy were performed on a Zeiss LSM 780 (Zeiss, 63× oil objective, 1.4 numerical aperture, with differential interference contrast prism). *Z*-stack images were obtained to capture entire ciliate cells and fluorescence images of FISH probe and DAPI signals were projected into 2D for visualization. Cell counting was performed using a Axio Imager 2 microscope (Zeiss) in randomly selected fields of view (40× objective, grid length = 312.5 μm) on polycarbonate filters (3-μm pore size, 32-mm effective filter diameter; Merck Millipore) onto which 0.5 l PFA-fixed lake water was filtered.

For light microscopy, live ciliates were picked from Lake Zug water (May 2019, 189 m) and prepared for live ciliate imaging using light microscopy as previously described^56^ on an Axio Imager 2 microscope (Zeiss). For image acquisition and processing, Zeiss ZEN (blue edition) 2.3 was used.

### Scanning electron microscopy

Ciliates sampled in February 2020 were individually picked under a binocular microscope, and washed in droplets of sterile-filtered lake water. Washed ciliates were then transferred into approximately 200 μl fixative on top of a polylysine-coated silicon wafer (0.1 mg ml^−1^ poly-l-lysine for 10 min at room temperature) and fixed for 1 h at room temperature. The fixative contained 2.5% glutaraldehyde (v/v, electron microscopy grade) in PHEM buffer^57^ (pH 7.4). Fixed ciliates attached to the silicon wafer were dehydrated in an ethanol series (30%, 50%, 70%, 80%, 96% and 100%) before automated critical-point drying (EM CPD300, Leica). Scanning electron microscopy was performed on a Quanta FEG 250 (FEI). Images were obtained using FEI xTM v.6.3.6.3123 at an acceleration voltage of 2 kV under high vacuum conditions and were captured using an Everhart–Thornley secondary electron detector. The image represents an integrated and drift corrected array of 128 images captured with a dwell time of 50 ns.

### Single-ciliate PCR

Ciliates were picked from Lake Zug water (189 m, 2019) under the binocular microscope with a glass micropipette, and subsequently washed twice in drops of sterile nuclease-free water (Ambion) before being transferred into lysis buffer. DNA was extracted using MasterPure DNA purification kit (Ambion) following the manufacturer’s instructions with a final elution in 1× TE buffer (25 μl). Overall, DNA was extracted from four individual ciliates (S1–S4), five (C5) and ten pooled ciliates (C10) as well as no ciliate (control). 16S (‘*Ca*. A. ciliaticola’) and 18S rRNA gene sequences (ciliate host) were then separately amplified by PCR using primer pairs eub62A3_29F/_1547R and cil_384F/_1147R. PCR reactions (20 μl) with ‘*Ca*. A. ciliaticola’-specific primers (eub62A3_29F/_1547R) were performed as described in ‘Clone library construction and Sanger sequencing’ with following modifications: 5 μl template, 58 °C annealing temperature and 40 amplification cycles. PCR with ciliate-specific primers (cil_384F/_1147R) was performed analogously with following modifications: 55 °C annealing temperature, 50 s elongation and 35 amplification cycles. The PCR reactions with primer pairs eub62A3_29F/_1547R were further amplified in a second round of PCR (under the same conditions) using 2 μl PCR reaction from the first round. For each PCR step, successful amplification of products was checked using gel electrophoresis as described in Supplementary Methods. PCR reactions were subsequently purified using QIAquick PCR purification Kit (Qiagen) according to the manufacturer’s instructions with a final elution in sterile nuclease-free water (25 μl) (Ambion). Purified PCR reactions were subsequently sequenced using Sanger sequencing and processed as described in ‘Clone library construction and Sanger sequencing’ with the following modifications: primers eub62A3_29F, eub62A3_1547R (annealing temperature 58 °C) or cil_384F, cil_1147R (annealing temperature 55 °C). Two of the single-ciliate DNA extracts amplified with the endosymbiont-specific primers either showed a faint (S4) or no (S2) band and were not sequenced.

### Nucleic acid extraction from lake water

Bulk DNA and RNA extraction as well as metagenome and metatranscriptome sequencing of lake water samples collected in 2016 have previously been described^46^. For samples from 2018, filters were purged of RNAlater, briefly rinsed with nuclease-free water and removed from the Sterivex cartridge. RNA and DNA was then extracted from separate filters using PowerWater RNA or DNA isolation kits (MoBio Laboratories) according to the manufacturer’s instructions.

### Metagenome and bulk metatranscriptome sequencing

For metagenomic sequencing, DNA libraries were prepared as recommended by the NEBNext Ultra II FS DNA Library Prep Kit for Illumina (New England Biolabs). Sequencing-by-synthesis was performed on the Illumina HiSeq2500 sequencer (Illumina Inc.) with the 2 × 250-bp read mode. For metatranscriptomic sequencing, rRNA was removed (Ribo-Zero rRNA Removal Kit for bacteria (Illumina)) and an RNA-sequencing library was prepared according to the NEBNext Ultra II Directional RNA Library Prep Kit for Illumina (New England Biolabs). Sequencing-by-synthesis was performed on the Illumina HiSeq3000 sequencer (Illumina) with the 1 × 150-bp read mode. Library preparation and sequencing was performed by the Max Planck Genome Centre Cologne (http://mpgc.mpipz.mpg.de/home/). Detailed information for each metagenomic and metatranscriptomic dataset can be found in Supplementary Table 4.

### Genome assembly and finishing

The genome of ‘*Ca*. A. ciliaticola’ was reconstructed from a metagenomic dataset sampled in 2018, as follows: metagenomic reads (MG_18_C) were trimmed using Trimmomatic^58^ v.0.32 as previously described^46^ and assembled using metaSPAdes^59^ v.3.13.0 and *k*-mer lengths of 21, 33, 55, 77, 99 and 127. From this assembly, contigs with high similarity to the previously reconstructed ‘*Ca*. A. ciliaticola’ genome from the metagenome samples from 2016 (further details are provided in Supplementary Methods) were identified by blastn (identity >95%) and metagenomic reads were mapped back to the contigs using BBmap^60^ v.35.43 (minid = 0.98). Mapped reads were subsequently reassembled using SPAdes v.3.13.0 with mismatch corrector and coverage threshold enabled (--careful --cov-cutoff 60), resulting in the assembly of a single contig (292,647 bp) that was circularized by trimming the identical overlapping ends (127 bp) giving rise to the closed genome (292,520 bp). The start position was set in an intergenic spacer region near the maximum of the GC disparity curve generated with oriFinder^61^ v.1.0. The two independently assembled circular metagenome-assembled genomes (from 2016 and 2018 metagenomes (Supplementary Methods)) shared 99.99% identity. For all subsequent analyses, the genome reconstructed from the 2018 dataset was used owing to higher coverage.

### Genome annotation and comparative analyses

Genome annotation was performed using a modified version of Prokka^62^ v.1.13.3 to allow annotation of genes that overlap with tRNA genes. The annotation of key metabolic genes was manually inspected and refined using searches against NCBI non-redundant protein or conserved domain database^63^. Transmembrane transporters were predicted and classified using the Transporter Automatic Annotation Pipeline web service^64^ and the Transporter Classification Database^65^. Pseudogenes were predicted using pseudo finder^66^ v.0.11 and standard settings. Circular genome maps were created using DNAplotter^67^ v.18.1.0.

For comparative analyses, protein-coding CDS encoded in the genomes of insect endosymbionts (*C. ruddii* PV, AP009180.1 and *B. aphidicola* BCc, CP000263.1), mitochondrion of *J. libera* (NC_021127) and a free-living relative of ‘*Ca*. A. ciliaticola’ (*L. clemsonensis*, CP016397.1) were downloaded from NCBI GenBank. Additionally, protein-coding CDS of the ciliate endosymbiont *C. taeniospiralis* (PGGB00000000.1) were obtained using Prokka annotation. Classification of functional categories was performed using the eggNOG-mapper v.1 web service^68^ with mapping mode DIAMOND and standard settings. The classification of the functional category C (energy production and conversion) for ‘*Ca*. A. ciliaticola’ was modified to also include *norB* and *nirK*, which were grouped by eggNOG into different categories (Q and P, respectively).

Multiple sequence alignment of ‘*Ca*. A. ciliaticola’ and other plastidic and bacterial ATP/ADP translocases was generated using MuscleWS^69^ v.3.8.31 with default settings implemented in Jalview^70^ v.2.11.1.0.

### Metatranscriptomic analyses of bulk water samples

Raw metatranscriptomic Illumina reads trimming and removal of rRNA sequences was performed as previously described^46^. Non-rRNA reads were then mapped to the genome of ‘*Ca*. A. ciliaticola’ using Bowtie2^71^ v.2.2.1.0 and standard parameters. Sorted and indexed BAM files were generated using samtools^72^ v.0.1.19 and transcripts per feature (based on the Prokka annotation) were quantified using EDGE-pro^73^ v.1.3.1 and standard settings. Gene transcription was subsequently quantified as transcripts per million^74^ (TPM) using the formula:

$${{\rm{T}}{\rm{P}}{\rm{M}}}_{{\rm{i}}}=\frac{{c}_{i}}{{l}_{i}}\times \frac{1}{{\sum }_{j}\frac{{c}_{i}}{{l}_{i}}}\times {10}^{6}$$

to assign each feature (*i*) a TPM value, in which *c* = feature count, *l* = length (in kb) and *j* = all features.

Read coverage visualization and plotting was performed using pyGenomeTracks^75^ (average coverage over 100-bp bins) implemented in deepTools2^76^ v.3.2.0.

### Phylogenetic analyses

The full-length 16S rRNA gene sequence was retrieved from the circular metagenome-assembled genome of ‘*Ca*. A. ciliaticola’ using RNAmmer^77^ v.1.5, aligned using the SILVA incremental aligner^49^ (SINA 1.2.11) and imported to the SILVA SSU NR99 database^45^ (release 132) using ARB^48^ v.6.1. Additional closely related 16S rRNA gene sequences were identified by BLASTN in the NCBI non-redundant nucleotide database and JGI IMG/M 16S rRNA Public Assembled Metagenomes (retrieved July 2018) and also imported into ARB. A maximum-likelihood phylogenetic tree of 16S rRNA gene sequences was calculated using RAxML^78^ v.8.2.8 integrated in ARB with the GAMMA model of rate heterogeneity and the GTR substitution model with 100 bootstraps. The alignment was not constrained by a weighting mask or filter. For the complete tree shown in Extended Data Fig. 4, additional ‘*Ca*. A. ciliaticola’ sequences obtained from a clone library and individual single ciliates were added to the tree using the Parsimony ‘Quick add’ algorithm implemented in ARB.

For the ciliate phylogeny, sequences obtained from Sanger sequencing of picked ciliates were added to the EukRef-Ciliphora^30^ Plagiopylea subgroup alignment using MAFFT^79^ online service version 7 (argument:--addfragments). An additional metagenome-assembled full-length 18S rRNA gene sequence assigned to Plagiopylea was obtained using phyloFlash^80^ v.3.0 from one of the Lake Zug metagenomes (MG_18_C) and also added to the alignment (argument:--add). A phylogenetic tree was calculated using IQ-TREE webserver (http://iqtree.cibiv.univie.ac.at) running IQ-TREE^81^ 1.6.11 with default settings and automatic substitution model selection (best-fit model: TIM2+F+I+G4). Phylogenetic trees were visualized using the Interactive Tree of Life v.4 web service^82^.

For the ATP/ADP translocase phylogenetic tree, amino acid sequences were retrieved from NCBI RefSeq (250 top hits) and NCBI nr (15 top hits) (both accessed June 2019) using NCBI blastp web-service^83^ with the amino acid sequence of ATP/ADP translocase sequence of ‘*Ca*. A. ciliaticola’ (ESZ_00147) as query. Additional amino acid sequences of characterized nucleotide transporters listed in Supplementary Table 8 were also added. After removal of duplicates, sequences were clustered at 95% identity using usearch^84^ v.8.0.1623 and aligned using MUSCLE^69^ v.3.8.31. Phylogenetic tree construction using IQ-TREE (best-fit model: LG+F+I+G4) and visualization was performed as described for the 18S rRNA gene phylogenetic tree.

### Incubation experiments

Incubation experiments were performed to provide experimental evidence for the denitrifying activity linked to the ciliate host. Three incubations were set up that contained (a) no ciliates (filtered fraction), (b) lake water that was enriched in ciliates (enriched fraction) and (c) bulk lake water. For these experiments, lake water was size-fractionated using a 10-μm polycarbonate filter (Merck Millipore) under N_2_ atmosphere in a glove bag at 12 °C. Enriched and filtered fractions were obtained by gravity filtration of 0.5 l water until 0.25 l supernatant was left. Thus, in the enriched fraction, ciliates from 0.5 l lake water were concentrated in 0.25 l lake water. In the filtered fraction, organisms larger than 10 μm (including ciliates) were filtered out. Both the enriched water (plus filter) and the filtered water were transferred to separate serum bottles (no headspace) and closed with butyl rubber stoppers. For bulk incubations, unfiltered water was directly filled into 250 ml serum bottles under N_2_ atmosphere.

Denitrification potential was assessed by measuring the production of ^30^N_2_ over time in ^15^N-nitrite and ^15^N-nitrate amended incubations by isotope ratio mass spectrometry (Isoprime Precision running ionOS v.4.04, Elementar). A 30 ml helium headspace was set for the 250 ml serum bottles and the water was degassed with helium for 10 min to ensure anoxic conditions and reduce N_2_ background. A ^15^N-labelled mixture of nitrate and nitrite (20 μM and 5 μM final concentration, respectively; Sigma Aldrich) was supplied at a 99% labelling percentage and the water was incubated for a total of 30 h at 4 °C in the dark. Subsamples of the headspace were taken at regular time intervals during the incubation by withdrawing 3 ml of the gaseous headspace and simultaneously replacing the removed volume by helium. This gas sample was transferred into 12 ml Exetainers (LabCo) that were pre-filled with helium-degassed water and stored until analysis. ^30^N_2_ in the gas samples was measured on an isotope ratio mass spectrometer, and denitrification rates were calculated from the slope of the linear increase of ^30^N_2_ in the headspace over the time course of the experiments. The rate of ^30^N_2_ production was corrected for dilution of the headspace introduced by subsampling and by the measured total ^15^N labelling percentage. ‘*Ca*. A. ciliaticola’-containing ciliate abundance in the different incubation bottles was assessed by microscopic counts after cell fixation, FISH hybridization (eub62A3_813) and DAPI staining as described in ‘Double-labelled oligonucleotide probe fluorescence in situ hybridization and microscopy’.

### Statistics and reproducibility

No statistical methods were used to predetermine sample size and experiments were not randomized. The investigators were not blinded to allocation during experiments and outcome assessment.

In Fig. 1a, the scanning electron microscopy image is a representative of *n* = 6 recorded images that were obtained from 1 experiment. In Fig 1b, the differential interference contrast image is a representative of *n* = 6 recorded images that were obtained from 1 experiment.

In Fig. 2c, Extended Data Fig. 2i. FISH fluorescence images (eub62A3_813 probe) are representative of *n* = 33 recorded images that were obtained from 5 independent experiments of 3 biological replicate samples.

In Fig. 2c, Extended Data Fig. 2f, h, j. DAPI fluorescence images are representative of *n* = 21 recorded images that were obtained from 5 independent experiments of 3 biological replicate samples.

In Extended Data Fig. 2a, the FISH fluorescence image (Arch915 probe) is representative of *n* = 6 images that were obtained from 1 experiment. In Extended Data Fig. 2b, d, F_420_ autofluorescence images are representative of *n* = 11 recorded images that were obtained from 3 independent experiments of 1 sample. In Extended Data Fig. 2c, g, FISH fluorescence images (EUB-I probe) are representative of *n* = 15 images obtained from 3 independent experiments of 2 biological replicate samples. In Extended Data Fig. 2e, the FISH fluorescence image (NON338 probe) is representative of *n* = 15 recorded images that were obtained from 3 independent experiments of 2 biological replicate samples.

In Extended Data Fig. 5a, each of the 6 FISH fluorescence images (eub62A3_813 probe) is representative of *n* = 3 images from 1 experiment.

For the fluorescence images shown, the number of analysed cells was typically much higher (*n* > 100) than the ones that were eventually recorded.

### Reporting summary

Further information on research design is available in the Nature Research Reporting Summary linked to this paper.

## Online content

Any methods, additional references, Nature Research reporting summaries, source data, extended data, supplementary information, acknowledgements, peer review information; details of author contributions and competing interests; and statements of data and code availability are available at 10.1038/s41586-021-03297-6.

## Supplementary information

Supplementary Information This file contains Supplementary Methods, Supplementary Tables 1–7, Supplementary Discussion, Supplementary Figure 1 and Supplementary References.

Reporting Summary

Supplementary Table 8 This file contains Supplementary Table 8, which lists characterized nucleotide transporters used for Extended Data Fig. 9.

Supplementary Table 9 This file contains Supplementary Table 9, which lists the number of genes assigned to COG functional categories encoded in the ‘*Ca*. A. ciliaticola’ and other genomes presented in Fig. 2c.

Supplementary Table 10 This file contains Supplementary Table 10, which lists transcripts (derived from ‘*Ca*. A. ciliaticola’-containing single ciliate transcriptomes) identified by homology searches against mitochondrially encoded reference sequences of ciliates.

Supplementary Table 11 This file contains Supplementary Table 11, which lists transcripts (derived from ‘*Ca*. A. ciliaticola’-containing single ciliate transcriptomes) putatively involved in hydrogenosomal and glycolytic metabolism of the ‘*Ca*. A. ciliaticola’-containing ciliate.

Supplementary Table 12 This file contains Supplementary Table 12, which lists FeFe-hydrogenase transcripts identified in ‘*Ca*. A. ciliaticola’-containing single ciliate transcriptomes.

Supplementary Table 13 This file contains Supplementary Table 13, which lists the number of small subunit rRNA reads assigned to a given taxon (as reported by the tool phyloFlash) for four ciliate transcriptome libraries (CT_01-04; Supplementary Table 4). Taxon names related to the plagiopylean host and ‘*Ca*. A. ciliaticola’ (eub62A3) were highlighted in bold.

Supplementary Video 1 This file contains a Supplementary Video 1, which shows a video of a living ciliate from Lake Zug (sampled in May 2019 from 189 m depth) observed using light microscopy.

Supplementary Video 2 This file contains a Supplementary Video 2, which shows a video of the two-capillary experimental setup which allows for simultaneous oxygen measurements and ciliate tracking. The upper capillary indicates the oxygen concentration where bright purple represents anoxic conditions and dark green oxic conditions. The moving white line indicates the 10 % oxycline. The lower capillary shows the position and movement of the ciliates relative to the changing oxygen gradients. Shown are short intervals (~30 seconds) recorded approximately every five minutes over the course of 30 minutes. See Supplementary Methods for further information on the image processing.

## Data Availability

The annotated genome of ‘*Ca*. A. ciliaticola’ has been deposited at the European Nucleotide Archive (ENA) under the BioProject PRJEB27314 (accession number LR794158). Small-subunit rRNA gene sequences of ‘*Ca*. A. ciliaticola’ and the plagiopylean host have been deposited at ENA under BioProject PRJEB27314 and accession numbers LR798074–LR798089. Raw metagenomic and metatranscriptomic sequencing data of bulk water samples as well as single-ciliate transcriptomes have been deposited at ENA under BioProject PRJEB36502 using the data brokerage service of the German Federation for Biological Data (GFBio)^85^. Metatranscriptomic sequencing data obtained in the year 2016 have previously been deposited at the Sequence Read Archive under BioProject PRJNA401219. Publicly available sequences used for phylogenetic trees are available under their respective accession codes at the SILVA rRNA database (http://www.arb-silva.de/) (Fig. 2e, Extended Data Fig. 4), JGI Integrated Microbial Genomes and Microbiomes database (http://img.jgi.doe.gov/) (Fig. 2e), EukRef-Ciliophora database (https://github.com/eukref/curation) (Fig. 2f), orthoDB (http://www.orthodb.org/) (Extended Data Fig. 6) and NCBI protein database (http://www.ncbi.nlm.nih.gov/protein/) (Extended Data Figs. 9, 10). Genomes used for comparative analyses are available under their respective accession codes at NCBI (http://www.ncbi.nlm.nih.gov/genome/) (Fig. 2b, c). Transporter classification information (Supplementary Table 6) can be found at the Transporter Classification Database (http://www.tcdb.org/). Source data are provided with this paper.

## Source data

Source Data Fig. 2 Source Data Fig. 3 Source Data Extended Data Fig. 1 Source Data Extended Data Fig. 5 Source Data Extended Data Fig. 7 Source Data Extended Data Fig. 8 Source Data Extended Data Fig. 10
