# Supplementary material for: Anaerobic endosymbiont generates energy for ciliate host by denitrification
Source: Nature. 2021 Mar 3;591(7850):445–50. doi: 10.1038/s41586-021-03297-6 (PMC7969357; doi:10.1038/s41586-021-03297-6)
Supplement: Supplementary file 1 — This file contains Supplementary Methods, Supplementary Tables 1–7, Supplementary Discussion, Supplementary Figure 1 and Supplementary References. [file 41586_2021_3297_MOESM1_ESM.docx]

Supplementary Information

This file contains Supplementary Methods, Supplementary Tables 1–7, Supplementary Discussion, Supplementary Figure 1 and Supplementary References.

**Table of Contents**

Supplementary Methods…………. 02

Supplementary Tables…………… 09

Supplementary Discussion………. 14

Supplementary Figure……………. 22

Supplementary References……… 23

**Supplementary Methods**

**Initial ‘*Ca*. A. ciliaticola’ genome re-construction.** Two genomes were independently reconstructed from metagenomic datasets sampled in 2016 and 2018. For the initial ‘*Ca*. A. ciliaticola’ genome reconstruction from metagenomes derived from water samples in 2016, raw metagenomic paired-end Illumina reads (2 x 250 bp) were trimmed as described in Methods. Three trimmed metagenomic datasets sampled in 2016 (MG_16_A, B, C) were co-assembled using metaSPAdes assembler^58^ 3.9.1 and k-mer lengths of 21,33,55,77,99,127. A single contig (289,063 bp) that represented almost the complete genome of ‘*Ca*. A. ciliaticola’ was then iteratively extended and polished as follows: combined metagenomic reads used for assembly were mapped to the contig using BBmap^59^ 35.43 and standard settings. Mapping paired-end reads were then reassembled with SPAdes 3.10.1 with the same k-mer lengths as before and mismatch corrector (--careful). Next, the mapping step with the combined metagenomic reads was repeated using the longest contig from the first re-assembly as reference and the mapped reads were then assembled again using SPAdes 3.10.1 supplied with the longest contig from the previous assembly as trusted contig. This process of read mapping and assembly was repeated seven times and resulted in a contig, which was extended by 3,457 bp compared to the original contig of 289,063 bp, where the sequences at both ends showed an identical overlap of 127 bp. The combined metagenomic reads were mapped to this contig using BBmap and strict mapping settings (minid=0.95, maxindel=1000) and the mapped reads were assembled using SPAdes 3.10.1 with mismatch corrector enabled but without supplying a trusted contig. Finally, the longest contig from this assembly was then circularized by removing one overlapping end resulting in the initial “*Ca*. A. ciliaticola” genome. An additional genome of “*Ca*. A. ciliaticola”, which was used for all subsequent analyses due to higher coverage, was reconstructed as described in Methods. Both reconstructed genomes had the same length and were almost identical (40 mismatches out of 292,520 bp).

**Clone library construction.** The ‘*Ca*. Azoamicus’-specific PCR was performed using the Platinum *Taq* DNA polymerase kit (Thermo Fisher Scientific) and included 0.75 µl MgCl_2_ (50 mmol l^-1^), 2.5 µl *Taq* reaction buffer (10x), 2.5 µl deoxyribose nucleotide triphosphate mix (dNTPs, 2 mmol l^-1^), 0.12 µl eub62A3_29F and eub62A3_1547R primers (50 pmol µl^-1^; Biomers, Ulm, Germany), 2.5 µl bovine serum albumin (10 mg ml^-1^), 0.1 µl Platinum Taq DNA polymerase (10 U µl^-1^), 15.4 µl PCR water and 0.5 µl template. The PCR reaction proceeded as follows: initial denaturation (94 °C, 2 min), 35 cycles of denaturation (94 °C, 30 s), annealing (55.8 °C, 30 s), elongation (72 °C, 90 s) followed by final elongation (72 °C, 10 min). The resulting PCR product was purified using QIAquick PCR purification kit (Qiagen) and subsequently used as a template for a PCR reaction with the general bacterial primers 8F and 1492R. The PCR reaction and product purification was performed analogous as before with following modifications to the PCR reaction: 0.25 µl of each 8F and 1492R primers (50 pmol µl^-1^; Biomers), 2 µl template (purified specific PCR product), annealing temperature 55 °C.

Ligation and transformation of the purified PCR product (4 µl) obtained with primers 8F and 1492R into chemically-competent *Escherichia coli* TOP10 cells was done using the TOPO TA cloning kit (Thermo Fisher Scientific) according to manufacturer’s instructions. The transformed cell suspension (10 µl) was plated onto LB medium (10 g l^-1^ Tryptone, 5 g l^-1^ NaCl, 5 g l^-1^ Yeast extract, pH 7.0) agar (5 g l^-1^) plates containing kanamycin (50 µg ml^-1^) and 40 µl 5-bromo-4-chloro-3-indolyl-β-D-galactopyranoside (X-gal, 50 mg ml^-1^ in dimethylformamide) and incubated at 37 °C overnight. In total, 15 white colonies were picked with sterile toothpicks which were dipped in PCR master-mix (same as used for general PCR, using M13F and 1492R primer pair) to confirm correct insert orientation, stroked onto LB agar PCR master plates and added to 5 ml LB liquid medium (both containing 50 µg ml^-1^ kanamycin and incubated at 37 °C overnight). Correct insert orientation and size (PCR product ~1500 bp) was confirmed by agarose gel electrophoresis (1.5% in TAE buffer). 100 bp DNA ladder (New England Biolabs) served as marker. Based on this, five clones were chosen at random and the plasmids were purified according to manufacturer’s instructions from the corresponding liquid cultures using a PureLink Quick Plasmid Miniprep Kit (Thermo Fisher Scientific).

**Chemotaxis assays with ciliates.** Chemotaxis experiments were performed to follow the locomotion and tactical reaction of individual ciliates to oxygen exposure. For this purpose single ciliates were picked from lake water samples after enrichment by gravity flow through a 5 µm filter. Care was taken not to expose the ciliates to oxygen or temperature changes, therefore experiments were performed in a temperature-controlled room at 14°C and sample handling was done in a glove bag filled with dinitrogen gas. ~17 ciliates were collected in a droplet of sterile-filtered lake water and, subsequently, transferred into a glass capillary with 1.5 mm inner-diameter using capillary motion. The capillary with the ciliates was placed in an Exetainer (Labco) prefilled with degassed water. One end of the capillary was protruding from the Exetainer cap and was open to the oxic atmosphere (Extended Data Fig. 3). A second capillary was placed in approx. 4 mm distance to the first capillary and filled with 100 µg ml^-1^ oxygen-sensitive nanoparticles dissolved in sterile-filtered lake water for ratiometric luminescence imaging to monitor the oxygen diffusion in parallel. The nano-particles are composed of the oxygen-sensitive dye Platinum(II) meso-(2,3,4,5,6-pentafluoro)phenyl porphyrin (PtTFPP) and a reference dye Macrolex fluorescence yellow 10GN (MY), both immobilized in a polymer matrix^87,88^. The Exetainer with both capillaries was mounted onto a custom-build long-distance microscope setup equipped with a 7x magnification lens system (Optem Fusion) and a microscope camera (Grasshopper3, 5.0 MP, 15 FPS, SONY ICX625, RGB, FLIR). Nano-particle excitation and ciliate illumination was performed for 15 ms with a bright blue light emitting diode (LPS 3, ILA5150) through a semi-transparent mirror installed in the lens system. The locomotion of the ciliates as well as the development of the oxygen gradient was recorded over 10 minutes, whereby at 0, 5 and 10 minutes the movement was continuously recorded for 30 s at 2 Hz. The recorded color-images were decomposed into the red, green and blue channel, where the red and green channels were used for determination of oxygen concentrations and the blue channel was used for ciliate tracking.

Oxygen concentrations were determined by calculating the pixelwise ratio of the red emission by the oxygen-sensitive dye (PtTFPP) and the green emission of the reference dye (MY) in Matlab (Mathworks 2018b). Calibration was performed in the same setup using nano-particles dissolved in MQ water at five different oxygen concentrations. The resulting functional response of the red-to-green ratio to the oxygen concentrations was fitted using the Stern-Volmer equation. Ciliate position and size were determined using a tracking algorithm implemented in Matlab (Mathworks 2017b). Briefly, the average background was subtracted from the time-lapse recordings and ciliates were identified as spots with increased brightness. Ciliate area and position was determined by calculating the connected bright pixels with a minimum area of 31 µm^2^ (2 px x 2 px) to avoid tracking of noise.

**Single ciliate transcriptomics.** For single ciliate transcriptomics, lake water samples were obtained from the anoxic hypolimnion of Lake Zug (185 m, February 2020) as described for the other years. Ciliates were individually picked anoxically with a micropipette at 12°C in a glove bag filled with N_2_ and washed by subsequent transfers of the ciliate into PCR water droplets (4 transfers) while minimizing the volume for each transfer. After washing, 1 µl PCR water containing the ciliate was transferred into a 96-well PCR plate (Bio-Rad), frozen in liquid N_2_ and stored at -80°C. Three transcriptomic libraries from single ciliates (CT_01 – 03) and one library from four combined ciliates (CT_04) were generated with the QIAseq FX Single Cell RNA Library Kit (Qiagen) with either oligo-dT (library CT_01) or a mixture of random and oligo-dT priming (libraries CT_02 – 04) to target eukaryotic and prokaryotic transcripts (Supplementary Table 4). Amplification products were fragmented and an Illumina-compatible library was produced, followed by sequencing-by-synthesis on a HiSeq3000 (Illumina) with 2 x 150 bp paired end read mode.

Adapter removal and trimming of raw transcriptomic reads was performed using Trimmomatic^57^ 0.39 and parameters “TruSeq3-PE.fa:2:30:10:2:keepBothReads LEADING:3 TRAILING:3 SLIDINGWINDOW:4:10 MINLEN:75”.

Community profiling based on small subunit rRNA sequences of the trimmed transcriptomic reads was performed using phyloFlash^80^ v3.3b3 (parameters “-readlength 150 -readlimit 1000000 -almosteverything) and the SILVA database^77^ (release 138). An NTU table was generated using the utility script “phyloFlash_compare.pl'' supplied with phyloFlash. Only NTUs that had >3 read counts in any sample were retained.

Prior to assembly, ribosomal RNA reads were removed from the trimmed transcriptomic reads using SortMeRNA^89^ v2.1 and the prepackaged eight rRNA databases (silva‐bac‐16s‐id90, silva‐arc‐id95, silva‐euk‐18s‐id95, silva‐bac‐23s‐id98, silva‐arc‐23s‐id98, silva‐euk‐28s‐id98, rfam‐5s‐id98, rfam‐5.8s‐id98). Next, non-rRNA reads from the single ciliate libraries were taxonomically classified using Kaiju^90^ (version 1.7.3, default parameters) and the nr+euk database (released 25th May 2020) and standard parameters. Reads classified as being bacterial, archaeal or viral were removed while retaining reads classified as either eukaryal or unclassified. The taxonomically filtered reads from all four libraries were combined and co-assembled using rnaSPAdes^91^ (version 3.14.1, default parameters). ORF prediction and amino acid translation of assembled transcripts was performed using Transdecoder^92^ (version 5.5.0) either with the Ciliate nuclear code (parameters: '-G Ciliate, -m 70') or Protozoa mitochondrial code (parameters '-G Mitochondrial-Protozoan -m 30'). Transcriptome completeness was assessed using BUSCO^93^ (version 4.0.6, parameters: ' -m protein -l alveolata' ) based on the predicted ORFs by Transdecoder using the Ciliate nuclear code. Phylogenies were reconstructed using the inferred complete single-copy orthologs and corresponding BUSCO ortholog groups. Therefore, ortholog proteins of alveolates were downloaded via API from orthoDB^94^ (version 10.1) and combined with the corresponding ORF inferred from ciliate host sequencing. A multiple sequence alignment per BUSCO group was obtained using the MAFFT tool^95^ (Version 7.407, 'linsi‘ option). Afterwards, phylogenetic trees were reconstructed from alignments via IQ-TREE^96^ (Version 1.6.10, parameters:' -alrt 1000 -bb 1000') and the resulting phylogenies were rooted in FigTree^97^ (version 1.4.4) on the root branches inferred by the MAD method^98^ (version 2.2).

For identification of transcripts encoded by mitochondrial DNA (mtDNA), ORFs (amino acid) predicted using the Protozoa mitochondrial code from the taxonomically filtered assembly were used as a BLAST database for homology searches using BLASTP^83^ v2.9.0 with a set of reference amino acid sequences as query (e-value cutoff 10^-6^). The reference set included mtDNA-encoded protein sequences obtained from NCBI Proteins database (*Tetrahymena pyriformis*, AF160864.1; *Sterkiella historimuscorum*, JN383843.1; *Paramecium aurelia*, NC_001324.2) and ref. Lewis *et al*.^17^ *(Cyclidium porcatum*, *Metopus contortus*, *Metopus es*, *Metopus striatus*, *Nyctotherus ovalis*). From the resulting hits, sequences that shared >95% amino acid identity with protein sequences encoded by ‘*Ca*. A. ciliaticola’ genome were identified using BLASTP and removed, resulting in four remaining sequences. Best BLASTP hits (by e-value) with the final four sequences as queries were generated using the mtDNA reference sequence set or NCBI non-redundant protein database (NCBI-nr, accessed 06/2020) as databases. For taxonomic assignment of transcript ORFs, amino acid sequences were queried against NCBI-nr using BLASTP (10^-6^ e-value cut-off) and the taxonomic assignment (collapsed at phylum) using the top ten blast hits (by e-value) was performed using the last common ancestor algorithm implemented in MEGAN6^99^ 6.19.4 with standard settings.

For identification of nuclear encoded hydrogenosomal and glycolytic transcripts, ORFs (amino acid) predicted using the Ciliate nuclear code were used as a BLAST database for homology searches (e-value cut-off 10^-6^) using BLASTP with a set of reference amino acid sequences as query. The hydrogenosome proteome reference set included all protein sequences of predicted hydrogenosome proteomes from *Cyclidium porcatium*, *Metopus contortus* and *Plagiopyla frontata* published in ref.^17^. Hits that shared <30% identity and <50% query coverage with the hydrogenosome proteome reference set and hits that were homologous to ‘*Ca*. A. ciliaticola’ proteins (>90% identity and >50% query coverage) were identified with BLASTP searches and removed. Best BLASTP hits (by e-value) with the remaining 71 sequences as queries were generated using the hydrogenosome protein reference sequence set and NCBI-nr as database. Functional annotation using amino acid sequences was performed using the KEGG Automatic Annotation Server^100^ (KAAS; accessed 08/2020) with the single-directional best hit method and the representative gene data set for Eukaryotes (plus *Tetrahymena thermophila* and *Paramecium tetraurelia*). Taxonomic classification was performed as described above.

For the identification of putative Fe-only hydrogenase transcripts, a set of reference [FeFe] hydrogenase amino acid sequences from several protists (*Plagiopyla frontata*, *Cyclidium porcatum*, *Metopus contortus*, *Trichomonas vaginalis*, *Nyctotherus ovalis*) was used as query against the predicted ORFs for homology searches (cutoffs: e-value <10^-6^, identity >30%, query coverage >10%) using BLASTP. Hits that were homologous to ‘*Ca*. A. ciliaticola’ proteins (>95% identity) were identified with BLASTP searches and removed. Sequences were validated as being [FeFe] hydrogenase by searches against the Conserved Domain Database^101^ (CDD) v3.18 and HydDB^102^ (both accessed 08/2020). A sequence was classified as being [FeFe] hydrogenase if domain hits against CDD models FeFe_hydrog_A superfamily, Fe_hyd_SSU or Fe_hyd_lg_C were found. The remaining validated [FeFe] hydrogenase sequences were taxonomically classified as described above. Finally, validated hydrogenase sequences that were either taxonomically classified as Ciliophora or had a hydrogenase sequence of *N*. *ovalis* as top BLASTP hit (by e-value) were retained.

**Supplementary Tables**

**Supplementary Table 1.** **Abundance of eukaryotic cells and ‘*Ca*. A. ciliaticola’-containing ciliates in Lake Zug (2018).** Eukaryotic cells were identified based on visualization of a nucleus (using DAPI staining) and weak autofluorescent outlines. ‘*Ca*. A. ciliaticola’-containing ciliates were identified by FISH (probe eub62A3_813).

| **Depth (m)** | **Number of eukaryotic cells examined** | **Total eukaryotes (cells l^-1^)** | **‘*Ca*. A. ciliaticola’-containing eukaryotes (cells l^-1^)** | **‘*Ca*. A. ciliaticola’-containing eukaryotes (%)** |
| --- | --- | --- | --- | --- |
| 160 | 29 | 6,046 | 2,502 | 41 |
| 170 | 46 | 9,354 | 8,947 | 95 |
| 180 | 77 | 25,365 | 25,365 | 100 |

**Supplementary Table 2. PCR Primers.** List of PCR primers names, sequences and associated references.

| **Primer name** | **Length** | **Sequence** | **Reference** |
| --- | --- | --- | --- |
| eub62A3-29_F | 23 nt | 5’-AATTTGTGTATTTAAAATCGAAG-3’ | - |
| eub62A3-1547_R | 22 nt | 5’-AGTTAAATACTATAAGGAGGTG-3’ | - |
| 8F | 18 nt | 5’-AGAGTTTGATYMTGGCTC-3’ | Juretschko, et al. ^103^ |
| 1492R | 19 nt | 5’-GGYTACCTTGTTACGACTT-3’ | Loy, et al. ^104^ |
| M13F | 16 nt | 5’-GTAAAACGACGGCCAG-3’ | - |
| M13R | 17 nt | 5’-CAGGAAACAGCTATGAC-3’ | - |
| Cil_384F | 20 nt | 5’-YTBGATGGTAGTGTATTGGA-3’ | Dopheide, et al. ^28^ |
| Cil_1147R | 20 nt | 5’-GACGGTATCTRATCGTCTTT-3’ | Dopheide, et al. ^28^ |

**Supplementary Table 3. FISH probes.** Double labelled FISH probes (all labelled with Atto488 on 5’- and 3’- ends, except unlabelled eub62A3_813 competitors) used in this study.

| **Probe name** | **Specificity** | **Sequence** | **Reference** |
| --- | --- | --- | --- |
| EUB-I | Most *Bacteria* | 5’-GCTGCCTCCCGTAGGAGT-3’ | Amann, et al. ^105^ |
| Arch915 | Most *Archaea* | 5’-GTGCTCCCCCGCCAATTCCT-3’ | Amann, et al. ^106^ |
| eub62A3_813 | *Ca*. Azoamicus subgroup A and most members of subgroup B | 5’- CTAACAGCAAGTTTTCATCGTTTA -3’ | - |
| eub62A3_813 comp. 1 | - | 5’- CTAACAGCAAGTTCTCATCGTTTA -3’ | - |
| eub62A3_813 comp. 2 | - | 5’- CCAACAGCAAGTTCTCATCGTTTA -3’ | - |
| NON338 | - | 5’- ACTCCTACGGGAGGCAGC-3’ | Wallner, et al. ^107^ |

**Supplementary Table 4. Sequencing datasets.** Summary of all metagenomic, metatranscriptomic and single ciliate transcriptome datasets generated in this study.

| **ID** | **Type** | **Sequencing technology** | **No. of (paired-end) reads** | **Total sequenced (Gbp)** | **Sample origin and date** |
| --- | --- | --- | --- | --- | --- |
| MG_16_A | DNA | HiSeq2500  (2×250 bp) | 25,085,070 | 12.5 | Lake Zug water column (110m), September 2016 |
| MG_16_B | DNA | HiSeq2500  (2×250 bp) | 23,611,159 | 11.8 | Lake Zug water column (120m), September 2016 |
| MG_16_C | DNA | HiSeq2500  (2×250 bp) | 25,567,478 | 12.8 | Lake Zug water column (160m), September 2016 |
| MG_18_C | DNA | HiSeq2500  (2×250 bp) | 20,519,714 | 10.3 | Lake Zug water column (180m), October 2018 |
| MT_16_C | RNA | HiSeq3000  (1×150 bp) | 67,587,421 | 10.1 | Lake Zug water column (180m), September 2016 |
| MT_18_C | RNA | HiSeq3000  (1×150 bp) | 78,233,812 | 11.7 | Lake Zug water column (180m), October 2018 |
| CT_01 | RNA | HiSeq3000  (2×150 bp) | 58,096,064 | 17.4 | Single ciliate, Lake Zug water column (185m), February 2020 |
| CT_02 | RNA | HiSeq3000  (2×150 bp) | 51,273,853 | 15.4 | Single ciliate, Lake Zug water column (185m), February 2020 |
| CT_03 | RNA | HiSeq3000  (2×150 bp) | 51,569,739 | 15.5 | Single ciliate, Lake Zug water column (185m), February 2020 |
| CT_04 | RNA | HiSeq3000  (2×150 bp) | 43,848,712 | 13.2 | Four ciliates, Lake Zug water column (185m), February 2020 |

**Supplementary Table 5. Transcription of selected functional genes of ‘*Ca*. A. ciliaticola’.** Listed are functional genes involved in denitrification, electron transport chain, ATP generation/exchange, tricarboxylic acid cycle, transmembrane transport (see also Supplementary Table 6), protein export as well as [Fe-S] cluster and bis-molybdopterin guanine dinucleotide (bis-MGD) biosynthesis. Transcription was quantified as transcripts per million (TPM) in metatranscriptomes obtained from Lake Zug in 2016 from 160 m (MT_16_C) and in 2018 from 180 m (MT_18_C).

| **Name** | **Gene** | **Locus tag** | **Transcription (TPM)** | |
| --- | --- | --- | --- | --- |
|  |  |  | **2016** | **2018** |
| **Denitrification** | | | | |
| Nitrate reductase | *narG* | ESZ_00058 | 7,789 | 6,282 |
|  | *narH* | ESZ_00059 | 11,049 | 8,428 |
|  | *narJ* | ESZ_00060 | 4,839 | 4,511 |
|  | *narI* | ESZ_00061 | 6,466 | 3,627 |
| Nitrite reductase, copper-containing | *nirK* | ESZ_00224 | 22,878 | 16,772 |
| Nitric oxide reductase | *norB* | ESZ_00225 | 21,906 | 13,004 |
|  | *norC* | ESZ_00226 | 20,174 | 18,714 |
| Nitrous oxide reductase | *nosZ* | ESZ_00265 | 45,424 | 37,835 |
| Nitrate transporter | *narT* | ESZ_00062 | 5,035 | 3,926 |
| Nitrate/nitrite transporter | *narK* | ESZ_00057 | 9,360 | 7,261 |
| **Electron transport chain** | | | | |
| Cytochrome *bc*_1_ complex | *qcrA* | ESZ_00136 | 4,477 | 5,752 |
|  | *qcrB* | ESZ_00135 | 3,797 | 3,949 |
|  | *qcrC* | ESZ_00134 | 2,786 | 3,646 |
| NADH dehydrogenase | *nuoA* | ESZ_00349 | 3,645 | 1,805 |
|  | *nuoB* | ESZ_00348 | 6,427 | 4,119 |
|  | *nuoC* | ESZ_00347 | 4,259 | 4,211 |
|  | *nuoD* | ESZ_00346 | 5,794 | 7,584 |
|  | *nuoE* | ESZ_00345 | 5,094 | 4,692 |
|  | *nuoF* | ESZ_00344 | 5,750 | 4,769 |
|  | *nuoG* | ESZ_00343 | 3,855 | 5,574 |
|  | *nuoH* | ESZ_00342 | 3,701 | 4,852 |
|  | *nuoI* | ESZ_00341 | 4,112 | 7,551 |
|  | *nuoJ* | ESZ_00340 | 3,861 | 4,866 |
|  | *nuoK* | ESZ_00339 | 3,223 | 4,390 |
|  | *nuoL* | ESZ_00338 | 2,552 | 3,292 |
|  | *nuoM* | ESZ_00337 | 4,635 | 4,992 |
|  | *nuoN* | ESZ_00336 | 4,140 | 3,782 |
| **ATP synthesis and exchange** | | | | |
| F0F1 ATP synthase | *atpB* | ESZ_00046 | 13,608 | 9,168 |
|  | *atpE* | ESZ_00047 | 40,396 | 30,674 |
|  | *atpF* | ESZ_00048 | 45,761 | 48,635 |
|  | *atpH* | ESZ_00049 | 28,181 | 31,862 |
|  | *atpA* | ESZ_00050 | 32,861 | 33,381 |
|  | *atpG* | ESZ_00051 | 23,454 | 30,565 |
|  | *atpD* | ESZ_00052 | 30,397 | 22,795 |
|  | *atpC* | ESZ_00053 | 30,331 | 31,276 |
| ATP/ADP translocase | *tlcA* | ESZ_00147 | 21,683 | 26,477 |
| **Tricarboxylic acid cycle and related enzymes** | | | | |
| 2-oxoglutarate:ferredoxin oxidoreductase | *korA* | ESZ_00304 | 5,136 | 5,285 |
|  | *korB* | ESZ_00305 | 6,527 | 4,161 |
| Succinate--CoA ligase | *sucC* | ESZ_00312 | 2,983 | 1,741 |
|  | *sucD* | ESZ_00313 | 3,231 | 1,882 |
| Succinate dehydrogenase | *sdhB* | ESZ_00216 | 3,995 | 4,651 |
|  | *sdhA* | ESZ_00217 | 4,853 | 4,573 |
|  | *sdhD* | ESZ_00218 | 3,215 | 1,717 |
|  | *sdhC* | ESZ_00219 | 861 | 504 |
| Malate dehydrogenase | *mdh* | ESZ_00034 | 24,835 | 18,130 |
| **Transporters** | | | | |
| Mla phospholipid trafficking system | *mlaB* | ESZ_00171 | 263 | 219 |
|  | *mlaC* | ESZ_00172 | 373 | 698 |
|  | *mlaD* | ESZ_00173 | 396 | 962 |
|  | *mlaE* | ESZ_00174 | 809 | 1,125 |
|  | *mlaF* | ESZ_00175 | 1,279 | 1,944 |
|  | *mlaA* | ESZ_00247 | 345 | 88 |
| Tyrosine-specific transport protein | *tyrP_1* | ESZ_00008 | 1,184 | 1,219 |
|  | *tyrP_2* | ESZ_00157 | 428 | 571 |
|  | *tyrP_3* | ESZ_00168 | 966 | 730 |
| Molybdate ABC transporter | *modA* | ESZ_00293 | 278 | 78 |
|  | *modB* | ESZ_00294 | 908 | 390 |
|  | *modC* | ESZ_00295 | 1,025 | 451 |
| Putative malate transporter | *yflS* | ESZ_00033 | 18,385 | 10,659 |
| **Protein export** | | | | |
| Sec-independent protein translocase | *tatA* | ESZ_00230 | 2,033 | 759 |
|  | *tatC* | ESZ_00146 | 806 | 328 |
| Sec protein translocase | *secA* | ESZ_00246 | 1,044 | 970 |
|  | *secY* | ESZ_00148 | 2,296 | 2,576 |
|  | *secE* | ESZ_00327 | 1,249 | 1,629 |
|  | *secG* | ESZ_00274 | 1,469 | 2,088 |
|  | *secD* | ESZ_00091 | 351 | 561 |
|  | *secF* | ESZ_00090 | 108 | 334 |
| **[Fe-S] and bis-MGD biosynthesis** | | | | |
| Suf system | *sufU* | ESZ_00286 | 2,180 | 1,284 |
|  | *sufS* | ESZ_00287 | 2,010 | 1,779 |
|  | *sufD* | ESZ_00288 | 186 | 167 |
|  | *sufC* | ESZ_00289 | 810 | 852 |
|  | *sufB* | ESZ_00290 | 1,250 | 826 |
| MGD biosynthesis | *moaA* | ESZ_00063 | 1,887 | 2,212 |
|  | *moaC/moaB* | ESZ_00064 | 3,075 | 3,165 |
|  | *moeA* | ESZ_00065 | 1,506 | 1,316 |
|  | *moaD* | ESZ_00066 | 78 | 358 |
|  | *moaE* | ESZ_00067 | 290 | 755 |
|  | *mobA* | ESZ_00068 | 471 | 551 |
|  | moeB | ESZ_00296 | 8,059 | 2,795 |

**Supplementary Table 6.** **Additional predicted transmembrane transporter genes not shown in Supplementary Table 5.** Transporters were classified using the Transport Classification (TC) system. Transcription was quantified as transcripts per million (TPM) in bulk metatranscriptomes obtained from Lake Zug in 2016 from 160 m (MT_16_C) and in 2018 from 180 m (MT_18_C).

| **Locus tag** | **Name** | **Gene** | **TC#** | **Transcription (TPM)** | |
| --- | --- | --- | --- | --- | --- |
|  |  |  |  | **2016** | **2018** |
| ESZ_00070 | K(+)-insensitive pyrophosphate-energized proton pump | *hppA* | 3.A.10 | 1,218 | 1486 |
| ESZ_00104 | Na(+)/H(+) antiporter NhaP | *nhaP* | 2.A.36 | 433 | 244 |
| ESZ_00105 | Glutamate/gamma-aminobutyrate antiporter | *gadC* | 2.A.3 | 729 | 363 |
| ESZ_00192 | S-adenosylmethionine/S-adenosylhomocysteine transporter | *-* | 2.A.7 | 249 | 388 |
| ESZ_00261 | Nitrous oxide reductase family maturation NosY | *nosY* | 3.A.1 | 979 | 1,200 |
| ESZ_00262 | Nitrous oxide reductase family maturation NosF | *nosF* | 3.A.1 | 2,956 | 4,963 |
| ESZ_00263 | Nitrous oxide reductase family maturation NosD | *nosD* | 3.A.1 | 3,372 | 4,975 |
| ESZ_00281 | Na(+)/H(+) antiporter NhaD | *nhaD* | 2.A.62 | 117 | 197 |
| ESZ_00283 | Putative polyketide transporter YadH | *yadH* | 3.A.1 | 604 | 423 |
| ESZ_00284 | Putative polyketide transporter YadG | *yadG* | 3.A.1 | 1,949 | 1,874 |
| ESZ_00335 | Putative permease PerM | *perM* | 2.A.86 | 652 | 869 |

**Supplementary Table 7. Nucleotides transporters (NTTs) with confirmed ATP/ADP translocase activity.** Listed are accession codes, gene names, organisms and substrate specificities as reported in the associated references.

| **Accession** | **Gene** | **Organism** | **Substrates** | **Reference** |
| --- | --- | --- | --- | --- |
| CAE46506 | PamNTT1 | *‘Ca*. Protochlamydia amoebophila’ | ATP/ADP | Haferkamp, et al. ^108^ |
| CAB39534 | CtNTT1 | *Chlamydia trachomatis* | ATP/ADP/NAD | Tjaden, et al. ^109^ |
| WP_013942850 | SnNTT1 | *Simkania negevensis* | ATP/ADP | Knab, et al. ^110^ |
| WP_004599717 | RpNTT1 | *Rickettsia prowazekii* | ATP/ADP | Audia and Winkler ^111^ |
| WP_012778542 | LaNTT1 | *Liberibacter asiaticus* | ATP/ADP | Vahling, et al. ^112^ |
| CAD29686 | CcNTT | *Caedimonas varicaedens* | ATP/ADP | Linka, et al. ^113^ |
| AAM80566 | HoNTT | *Holospora obtusa* | ATP/ADP | Linka, et al. ^113^ |
| NP_001274794 | StNTT1 | *Solanum tuberosum* | ATP/ADP | Tjaden, et al. ^114^ |
| CAC80882 | GsNTT1 | *Galdieria sulphuraria* | ATP/ADP | Linka, et al. ^113^ |
| CAA89201 | AtNTT1 | *Arabidopsis thaliana* | ATP/ADP | Neuhaus, et al. ^115^ |
| CAA64329 | AtNTT2 | *Arabidopsis thaliana* | ATP/ADP | Möhlmann, et al. ^116^ |
| ABW20407.1 | EcNTT1 | *Encephalitozoon cuniculi* | ATP/ADP | Tsaousis, et al. ^117^ |
| ABW20408.1 | EcNTT2 | *Encephalitozoon cuniculi* | ATP/ADP | Tsaousis, et al. ^117^ |
| ABW20409.1 | EcNTT3 | *Encephalitozoon cuniculi* | ATP/ADP | Tsaousis, et al. ^117^ |
| ABW20410.1 | EcNTT4 | *Encephalitozoon cuniculi* | ATP/ADP | Tsaousis, et al. ^117^ |

**Supplementary Discussion**

**Ciliate denitrification rates.** Deep anoxic lake water containing ciliates (189 m, May 2019) was incubated anaerobically with added ^15^N-nitrate and ^15^N-nitrite. The linear production of ^30^N_2_ over time in the bulk water provided direct evidence for ongoing denitrification at this depth (Extended Data Fig. 8). Active denitrification by ciliates was confirmed by comparing size-fractionated incubations with and without ciliates (see Methods). Incubations with no ciliates (<10 µm size fraction) showed a denitrification rate of ~60 nmol N l^-1^ d^-1^ whereas denitrification was enhanced in incubations containing ciliates (bulk water and >10 µm enriched water; up to 95 nmol N l^-1^ d^-1^; Fig. 3c). Crucially, the abundance of ‘*Ca.* A. ciliaticola’-containing ciliates was significantly correlated with the denitrification rates (R^2^ = 0.90, p = 0.003). The resulting average single ciliate denitrification rates were ~12±2 pmol N ciliate^-1^ day^-1^, which amounts to a volumetric rate of ~2.3 fmol N µm^-3^ day^-1^. For comparison, the volumetric denitrification rates reported for foraminifera^24^ are substantially lower and range between 0.002 and 0.05 fmol N µm^-3^ day^-1^.

**Estimated age and origin of the ‘*Ca*. A. ciliaticola’ endosymbiosis.** In order to approximate the age of the ‘*Ca*. A. ciliaticola’ endosymbiosis we used the 16S rRNA gene sequence divergence between the putatively symbiotic and non-symbiotic, free-living subgroups, assuming 1–2% 16S rRNA sequence divergence per 50 Ma^118^. The ‘*Ca*. Azoamicus’ subgroup A contains ‘*Ca*. A. ciliaticola’ and very closely (>98.5 %) related sequences, and we therefore consider all members of this subgroup to be obligately endosymbiotic. Assuming that the members of the ‘*Ca*. Azoamicus’ subgroup B are not yet endosymbionts, then the divergence between symbionts and non-symbionts occurred roughly at the split of subgroups A and B, which amounts to ~106–213 Mya ago. By this time, the ancient Ciliophora phylum had already diversified into many lineages, including anaerobic ones^119^. Note that this a conservative estimate, which could substantially increase if ‘*Ca*. Azoamicus’ subgroup B, or even the more distantly related NKB5 group, also contain endosymbiotic members.

Given that the ‘*Ca*. A. ciliaticola’ symbiosis is so ancient, its origin clearly predates the formation of the post-glacial Lake Zug (*~*10 ky old). Hence, the symbiosis must have evolved in an analogous location elsewhere. Nonetheless, many present-day freshwater bodies with permanently anoxic waters, steady availability of nitrate, and shallow oxygen and nitrate gradients, likely represent a suitable habitat for these anaerobic, nitrate-respiring eukaryotes.

In general, anaerobic eukaryotes still represent a vastly understudied group of organisms; owing, no doubt, to the difficulty of their collection and cultivation. Somewhat habitually, too, hydrogenosomal metabolism is often presumed for species thriving in the absence of oxygen. The discovery of the Azoamicus symbiosis suggests that the anaerobic metabolic capacities of these peculiar eukaryotes might be more diverse than we so far assumed.

**Genome-inferred metabolic potential of ‘*Ca*. A. ciliaticola’ related to carbon metabolism, chaperones and transporters.** The ‘*Ca*. A. ciliaticola’ genome encoded a subset of the TCA cycle genes (Fig. 3a, Supplementary Table 5). Two of these, the membrane-bound succinate dehydrogenase (*sdhABCD*) and malate dehydrogenase (mdh) likely operate in oxidative direction, producing reduced NADH and ubiquinol, respectively, which directly transfers electrons into the electron transport chain. This suggests that both succinate and malate might be important substrates for ‘*Ca*. A. ciliaticola’. On the other hand, 2-oxoglutarate oxidoreductase (korAB) and succinate--CoA ligase (*sucCD*) are decoupled from the ETC and could also work in the reverse direction, thus producing reduced ferredoxin (Fdx). Genes for ferredoxin were found to be encoded in the ‘*Ca.* A. ciliaticola’ genome and these [FeS] proteins might play a role in the cell as low potential electron donors. At this point, it is not clear which biosynthetic or respiratory complexes might utilize reduced ferredoxin, as the Rnf complex and other common Fdx-utilizing enzymes, were not encoded in the endosymbiont genome.

Further, the ‘*Ca*. A. ciliaticola’ genome contained genes encoding chaperone proteins GroES, GroEL, and DnaK, which were highly transcribed (GroEL belonging to the top 20 transcribed genes; Fig. 3b). Molecular chaperones tend to be highly expressed in obligate symbionts to ameliorate the effects of lower protein stability that results from their accumulation of deleterious mutations^120,121^. Genes involved in DNA repair (e.g. *mut*, *hol*, *rec*) or rod shape-determining genes (i.e. *ftsZ*, *ispA*, *mreB*, *rodA*) were absent from the ‘*Ca*. A. ciliaticola’ genome. These genes are also often missing from the extremely reduced genomes of insect endosymbionts^31,32^.

Due to the absence of most genes for the biosynthesis of essential cellular building blocks (i.e. nucleotides, amino acids, phospholipids, vitamins), ‘*Ca*. A. ciliaticola’ likely relies on its host to provision these metabolites. Yet, surprisingly few transporters were predicted in the endosymbiont genome (Supplementary Table 5 and 6). Three homologous genes predicted to encode for tryptophan/tyrosine-specific amino acid permeases could facilitate the uptake of aromatic amino acids. Additionally, the ‘*Ca*. A. ciliaticola’ genome contained a gene predicted to encode for a glutamate/gamma-aminobutyrate antiporter (*gadC*), which could be involved in the uptake of glutamate. However, it is not clear if indeed gamma-aminobutyrate (GABA) can be used by the endosymbiont since no genes for GABA metabolism were encoded in the genome. No other transporters for the uptake of other amino acids were identified in the ‘*Ca*. A. ciliaticola’ genome. Likewise, ‘*Ca*. A. ciliaticola’ did not encode genes for phospholipid biosynthesis. Interestingly, however, the genome encoded a complete Mla pathway (*mlaABCDEF*). This pathway, together with the likewise encoded putative polyketide drug exporter (*yadGH*), could be involved in the acquisition of phospholipids from the host, since it has been shown to be responsible for phospholipid trafficking between outer and inner membrane in Gram-negative bacteria^122-124^.

Several transporters appear to serve specific functions related to denitrification, such as the nitrate/nitrite transporters (*narK*), and the ABC transporters for molybdopterin uptake (*modABC*) and nitrous oxide reductase maturation (*nosDFY*). The encoded protein translocases (Sec and Tat-type) are likely needed for the translocation of putatively non-cytoplasmatic enzymes either directly or indirectly involved in respiratory denitrification (e.g. nitrous oxide reductase, nitrite reductase, cytochrome *c*_4_). Other predicted transporter-related genes included two sodium/proton antiporters (*nhaP/D*), a transporter related to Autoinducer-2 Exporter Family (*perM*), a H^+^-translocating pyrophosphatase (*hppA*) and a S-adenosylmethionine/S-adenosylhomocysteine transporter.

Nucleotide exchange and possibly uptake could be mediated by a nucleotide transporter (NTT) of the ATP:ADP Antiporter (AAA) family. These transporters exhibit specificity for one or several nucleotides or nucleoside-derived substrates^111^. In ‘*Ca*. A. ciliaticola’, the NTT transporter may mainly serve as an ATP/ADP translocase, albeit transport of other nucleotides cannot be excluded. For the plastidic NTT from *Arabidopsis thaliana* (AtNTT1), which functions as a ATP/ADP translocase, several charged amino-acid residues (K155, E245, E385, K527; counted on the basis of AtNTT1) have been shown to be important for nucleotide transport^86^. Specifically, K527 appears to be important for nucleoside triphosphate specificity^86^. All of these residues were also conserved in other functionally verified ATP/ADP translocase sequences and also in the NTT sequence of ‘*Ca*. A. ciliaticola’ (Extended Data Fig. 9). However, it should be noted that K527 was also conserved in NTTs with different or broader substrate specificity (Extended Data Fig. 9), such as NTTs of *Chlamydia trachomatis*^125^, *Rickettsia prowazekii*^111^ or ‘*Ca*. Protochlamydia amoebophila’^108^.

NTTs are a defining feature of an obligately intracellular lifestyle and all characterized single-domain NTTs (as opposed to NTT fusion proteins^39^) are either from parasites with reduced genomes or endosymbiotic organelles (e.g. plastids), where they allow the uptake of nucleotides and other host metabolites. NTTs are known to undergo horizontal transfer among bacteria as well as between bacteria and eukaryotes^39^. ATP/ADP translocases in particular are common among intracellular parasites and also some endosymbionts use this enzyme to obtain ATP from their host and complement their energy requirements^126^. ATP/ADP translocases are bidirectional and work along the concentration gradient^126,127^. In actively-metabolizing ‘*Ca*. A. ciliaticola’ cells, the intracellular ATP levels will be high and under such conditions the putative ATP/ADP translocase would export ATP out of the cell in exchange for ADP from the host cytoplasm.

**Microbial community composition and Ciliophora marker genes in single ciliate transcriptomes.** Seven ciliates were picked for the preparation of single ciliate transcriptome libraries. Their affiliation with Plagiopylea and the presence of ‘*Ca*. A. ciliaticola’ in their cells was confirmed by a taxonomic analysis of the small subunit rRNA gene (SSU) reads in the retrieved transcriptomes (Supplementary Table 13, CT_01–CT_04). In all four transcriptomes, we retrieved SSU reads belonging to the host and to ‘*Ca*. A. ciliaticola’ (classified as Plagiopylea and eub62A3, respectively). Overall, the combined relative abundance of SSU reads assigned to Plagiopylea and ‘*Ca*. A. ciliaticola’ was 9.75% (CT_01), 26.4% (CT_02), 3.2% (CT_03) and 21.4% (CT_04). In all libraries, >95% of all Ciliophora sequences were classified as belonging to Plagiopylea. The relative abundance of ‘*Ca*. A. ciliaticola’ varied between different libraries (1.7–7.5% of SSU sequences) but was generally among the top 10 observed taxa (except library CT_03). These results confirmed that the single cell transcriptomes were obtained from plagiopylean ciliates harboring ‘*Ca*. A. ciliaticola’. However, these single ciliate transcriptomes should, for all intents and purposes, be considered metatranscriptomes since a substantial fraction of the SSU reads appeared to be also associated with Gammaproteobacteria (mainly Orders Methylococcales, Burkholderiales) and also Eukaryota (mainly Cryptomonadales). Some of these organisms, e.g. the Methylococcales bacteria, have been shown to be prominent members of the indigenous microbial community in the anoxic hypolimnion of Lake Zug^128,129^. The detection of reads belonging to these organisms could be due to the presence of these organisms in the food vacuoles of the picked ciliates, which we also observed by microscopy (see Fig. 1 and Extended Data. Fig. 2). Alternatively, (some of) these organisms could constitute endo- or ecto-symbionts of the ciliate host.

Further, using Alveolata Benchmarking Universal Single-Copy Orthologs (BUSCOs; Complete or Fragmented, see Supplementary Methods) we estimated that *ca*. 55.6% of the host genome was covered. 19.9% of BUSCOs were full-length sequences (“BUSCO complete”), while only 4.1% were full-length sequences and found in multiple copies. To validate the taxonomic affiliation of retrieved BUSCO marker proteins with Ciliophora, phylogenies were reconstructed for three examples of proteins covering different cellular functions (Extended Data Fig. 6).

Our community analysis of the single ciliate transcriptomes further showed that methanogenic archaea, which often occur in hydrogenosome-bearing anaerobic ciliates, were virtually absent from the plagiopylean ciliate host. For three out of the four single ciliate transcriptomes we could not detect any archaeal 16S rRNA sequences; only in one transcriptome (CT_04) were we able to identify 8 reads taxonomically assigned to the genus *Methanoregula*. Although some species of *Methanoregula* indeed occur as endosymbionts of anaerobic ciliates^130^, the very low relative abundance of *Methanoregula* sequences (~0.004%) makes it highly unlikely that the same is the case for the ‘*Ca*. A. ciliaticola’-containing ciliate host.

**Detection of mitochondrial transcripts in single cell transcriptomes.** To investigate whether the plagiopylean host of ‘*Ca*. A. ciliaticola’ contains mitochondria, we compiled a reference set of protein sequences encoded by mitochondrial DNA (mtDNA) from eight different ciliate species (see Supplementary Methods for details). This reference set was used for homology searches against predicted open reading frames (ORFs) from a co-assembly of our four single ciliate transcriptomes. In total, we could identify four transcripts that were homologous to the mtDNA reference sequences (32–49% identity, see Supplementary Table 10). However, based on the subsequent taxonomic classification of the transcripts (see Supplementary Methods for details), none of these sequences appeared to be assigned to Ciliphora but rather to Bacteria or Streptophyta. Therefore, it seems more likely that the identified transcripts were of prokaryotic, rather than of mitochondrial origin. Hence, at this point, we have no evidence to suggest that the plagiopylean ciliate contains mitochondria.

**Detection of transcripts related to glycolytic and hydrogenosomal metabolism in single ciliate transcriptomes.** The presence of hydrogenosomes in the plagiopylean host of ‘*Ca*. A. ciliaticola’ was investigated using a similar approach as used for mitochondrially-encoded genes. As a reference dataset for homology searches, previously published sequences^17^ of hydrogenosomal proteins of the ciliates *Cyclidium procatum*, *Metopus contortus* and *Plagiopyla frontata* were used. In brief, the reference set broadly covered enzymes involved in carbon metabolism (cytosolic glycolysis, partial tricarboxylic acid cycle), Fe-only hydrogenase, protein translocation and processing, electron transport chain, oxidative phosphorylation, [FeS] cluster biosynthesis (Isc-type) and solute transmembrane transport.

Most importantly, we were able to retrieve three transcripts encoding a partial large subunit of the Fe-only hydrogenase. The sequences were very short (86–164 amino acids) but homology searches suggest that these sequences were likely of Ciliophora origin (Supplementary Table 12). We also found transcripts of the mitochondrial import inner membrane translocase TIM17 and of the mitochondrial solute carrier family proteins (Supplementary Table 11). Furthermore, we identified transcripts for six different enzymes of the cytosolic glycolysis pathway (Supplementary Table 11) for the generation of pyruvate (or malate), which can serve as substrates for hydrogenosomes^131^ (and ‘*Ca*. A. ciliaticola’). We could not identify homologs of the malic enzyme among the transcripts but we identified several transcripts of malate dehydrogenase homologs, which have been suggested to play a role in the hydrogenosomal malate metabolism of *Plagiopyla frontata*^17^.

Regarding the hydrogenosomal process of oxidative decarboxylation of pyruvate, we found transcripts encoding two subunits of the pyruvate dehydrogenase (PDH) complex (2-oxoglutarate dehydrogenase E1 component, dihydrolipoyl dehydrogenase E3 subunit). The PDH complex is commonly employed by hydrogenosome-containing ciliates^17^. Furthermore, we identified transcripts for the alpha subunit of succinyl-CoA synthetase that might be involved in ATP production via substrate-level phosphorylation, as it has been shown for hydrogenosomes of *Trichomonas vaginalis*^132^. However, we did not find transcripts encoding for pyruvate:ferredoxin oxidoreductase or pyruvate:NADP^+^ oxidoreductase, which are used in hydrogenosomes of some protists (such as *Tritrichomonas foetus*^133^ or *Euglena gracilis*^134^).

Overall, the results of our ciliate transcriptome analyses lend support to the speculation that hydrogenosome-like organelles are present in the ciliate host. However, further research is needed to clarify how similar are the hydrogenosomes of the ‘*Ca*. A. ciliaticola’-containing host to the hydrogenosomes of other ciliates, given the absence of transcripts for other proteins typically associated with hydrogenosomes, notably the Isc-type [FeS] cluster biosynthesis pathway.

**Supplementary Figure**


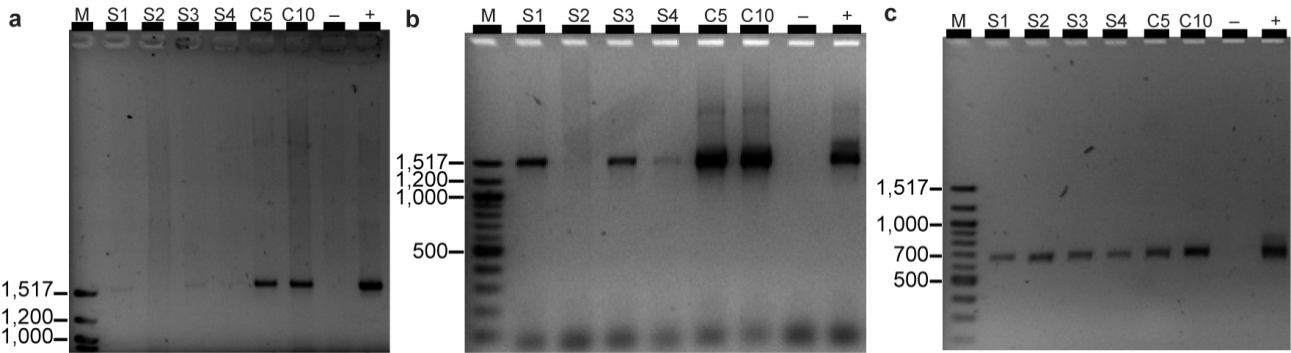


**Supplementary Figure 1. Agarose gels of PCR amplification of 16S and 18S rRNA genes from individual and pooled ciliates.** Agarose gels of PCR reactions (10 µl each) amplified with ‘*Ca*. A. ciliaticola’-specific 16S primer pair (eub62A3_29F, eub62A3_1547R; a,b) and ciliate-specific 18S primer pair (Cil_384F, Cil_1147R; c). DNA extracted from four individual ciliates (S1–S4) and pooled ciliates (C5, 5 ciliates; C10, 10 ciliates) picked from Lake Zug water in May 2019 from 189 m water depth served as templates (panels a and c). The 16S rRNA sequence of ‘*Ca*. A. ciliaticola’ was amplified in a second PCR reaction (b) where the PCR product from the first PCR reaction (a) was used. DNA extracted from bulk water (~2 l) sampled in September 2016 from 160 m water depth served as positive control (+) whereas the negative control (–) followed the same procedure used for the picked individual ciliates except that no ciliate was picked. A DNA ladder (100–1,517 bases) was used as marker (M) and marker fragment sizes are indicated for each panel as number of bases.

**Supplementary References**

87 Mistlberger, G. *et al.* Multifunctional magnetic optical sensor particles with tunable sizes for monitoring metabolic parameters and as a basis for nanotherapeutics. *Adv. Funct. Mater.* **20**, 1842–1851 (2010).

88 Koren, K., Brodersen, K. E., Jakobsen, S. L. & Kühl, M. Optical sensor nanoparticles in artificial sediments–a new tool to visualize O_2_ dynamics around the rhizome and roots of seagrasses. *Environ. Sci. Technol.* **49**, 2286–2292 (2015).

89 Kopylova, E., Noé, L. & Touzet, H. SortMeRNA: fast and accurate filtering of ribosomal RNAs in metatranscriptomic data. *Bioinformatics* **28**, 3211–3217 (2012).

90 Menzel, P., Ng, K. L. & Krogh, A. Fast and sensitive taxonomic classification for metagenomics with Kaiju. *Nat. Commun.* **7**, 11527 (2016).

91 Bushmanova, E., Antipov, D., Lapidus, A. & Prjibelski, A. D. rnaSPAdes: a *de novo* transcriptome assembler and its application to RNA-Seq data. *GigaScience* **8**, giz100 (2019).

92 Haas, B. J. *et al.* De novo transcript sequence reconstruction from RNA-seq using the Trinity platform for reference generation and analysis. *Nat. Protoc.* **8**, 1494–1512 (2013).

93 Simão, F. A., Waterhouse, R. M., Ioannidis, P., Kriventseva, E. V. & Zdobnov, E. M. BUSCO: assessing genome assembly and annotation completeness with single-copy orthologs. *Bioinformatics* **31**, 3210–3212 (2015).

94 Kriventseva, E. V. *et al.* OrthoDB v10: sampling the diversity of animal, plant, fungal, protist, bacterial and viral genomes for evolutionary and functional annotations of orthologs. *Nucleic Acids Res.* **47**, D807–D811 (2019).

95 Katoh, K. & Standley, D. M. MAFFT multiple sequence alignment software version 7: improvements in performance and usability. *Mol. Biol. Evol.* **30**, 772–780 (2013).

96 Nguyen, L.-T., Schmidt, H. A., Von Haeseler, A. & Minh, B. Q. IQ-TREE: a fast and effective stochastic algorithm for estimating maximum-likelihood phylogenies. *Mol. Biol. Evol.* **32**, 268–274 (2015).

97 Rambaut, A. *FigTree v1. 4* (http://tree.bio.ed.ac.uk/software/figtree/) (2012).

98 Tria, F. D. K., Landan, G. & Dagan, T. Phylogenetic rooting using minimal ancestor deviation. *Nat. Ecol. Evol.* **1**, 0193 (2017).

99 Huson, D. H., Auch, A. F., Qi, J. & Schuster, S. C. MEGAN analysis of metagenomic data. *Genome Res.* **17**, 377–386 (2007).

100 Moriya, Y., Itoh, M., Okuda, S., Yoshizawa, A. C. & Kanehisa, M. KAAS: an automatic genome annotation and pathway reconstruction server. *Nucleic Acids Res.* **35**, W182–W185 (2007).

101 Lu, S. *et al.* CDD/SPARCLE: the conserved domain database in 2020. *Nucleic Acids Res.* **48**, D265–D268 (2020).

102 Sondergaard, D., Pedersen, C. N. & Greening, C. HydDB: A web tool for hydrogenase classification and analysis. *Sci. Rep.* **6**, 34212 (2016).

103 Juretschko, S. *et al.* Combined Molecular and Conventional Analyses of Nitrifying Bacterium Diversity in Activated Sludge: *Nitrosococcus mobilis* and *Nitrospira*-Like Bacteria as Dominant Populations. *Appl. Environ. Microbiol.* **64**, 3042–3051 (1998).

104 Loy, A. *et al.* Oligonucleotide Microarray for 16S rRNA Gene-Based Detection of All Tecognized Lineages of Sulfate-Reducing Prokaryotes in the Environment. *Appl. Environ. Microbiol.* **68**, 5064–5081 (2002).

105 Amann, R. I. *et al.* Combination of 16S rRNA-targeted oligonucleotide probes with flow cytometry for analyzing mixed microbial populations. *Appl. Environ. Microbiol.* **56**, 1919–1925 (1990).

106 Amann, R. I., Krumholz, L. & Stahl, D. A. Fluorescent-oligonucleotide probing of whole cells for determinative, phylogenetic, and environmental studies in microbiology. *J. Bacteriol.* **172**, 762–770 (1990).

107 Wallner, G., Amann, R. & Beisker, W. Optimizing fluorescent in situ hybridization with rRNA‐targeted oligonucleotide probes for flow cytometric identification of microorganisms. *Cytometry* **14**, 136–143 (1993).

108 Haferkamp, I. *et al.* Tapping the nucleotide pool of the host: novel nucleotide carrier proteins of *Protochlamydia amoebophila*. *Mol. Microbiol.* **60**, 1534–1545 (2006).

109 Tjaden, J., Schwöppe, C., Möhlmann, T., Quick, P. W. & Neuhaus, H. E. Expression of a Plastidic ATP/ADP Transporter Gene in *Escherichia coli* Leads to a Functional Adenine Nucleotide Transport System in the Bacterial Cytoplasmic Membrane. *J. Biol. Chem.* **273**, 9630–9636 (1998).

110 Knab, S., Mushak, T. M., Schmitz-Esser, S., Horn, M. & Haferkamp, I. Nucleotide Parasitism by *Simkania negevensis* (*Chlamydiae*). *J. Bacteriol.* **193**, 225–235 (2011).

111 Audia, J. P. & Winkler, H. H. Study of the Five *Rickettsia prowazekii* Proteins Annotated as ATP/ADP Translocases (Tlc): Only Tlc1 Transports ATP/ADP, While Tlc4 and Tlc5 Transport Other Ribonucleotides. *J. Bacteriol.* **188**, 6261–6268 (2006).

112 Vahling, C. M., Duan, Y. & Lin, H. Characterization of an ATP Translocase Identified in the Destructive Plant Pathogen “*Candidatus* Liberibacter asiaticus”. *J. Bacteriol.* **192**, 834–840(2010).

113 Linka, N. *et al.* Phylogenetic relationships of non-mitochondrial nucleotide transport proteins in bacteria and eukaryotes. *Gene* **306**, 27–35 (2003).

114 Tjaden, J., Möhlmann, T., Kampfenkel, K. & Neuhaus, G. H. a. E. Altered plastidic ATP/ADP-transporter activity influences potato (*Solanum tuberosum* L.) tuber morphology, yield and composition of tuber starch. *Plant J.* **16**, 531–540 (1998).

115 Neuhaus, H. E., Thom, E., Möhlmann, T., Steup, M. & Kampfenkel, K. Characterization of a novel eukaryotic ATP/ADP translocator located in the plastid envelope of *Arabidopsis thaliana* L. *Plant J.* **11**, 73–82 (1997).

116 Möhlmann, T. *et al.* Occurrence of two plastidic ATP/ADP transporters in *Arabidopsis thaliana* L. *Eur. J. of Biochem.* **252**, 353–359 (1998).

117 Tsaousis, A. D. *et al.* A novel route for ATP acquisition by the remnant mitochondria of *Encephalitozoon cuniculi*. *Nature* **453**, 553–556 (2008).

118 Moran, N. A., Munson, M. A., Baumann, P. & Ishikawa, H. A molecular clock in endosymbiotic bacteria is calibrated using the insect hosts. *Proc. R. Soc. Lond.B.* **253**, 167–171 (1993).

119 Fernandes, N. M. & Schrago, C. G. A multigene timescale and diversification dynamics of Ciliophora evolution. *Mol. Phylogenet. Evol.* **139**, 106521(2019).

120 Baumann, P., Baumann, L. & Clark, M. A. Levels of *Buchnera aphidicola* Chaperonin GroEL During Growth of the Aphid *Schizaphis graminum*. *Curr. Microbiol.* **32**, 279–285 (1996).

121 Fares, M. A., Ruiz-González, M. X., Moya, A., Elena, S. F. & Barrio, E. Endosymbiotic bacteria: groEL buffers against deleterious mutations. *Nature* **417**, 398 (2002).

122 Babu, M. *et al.* Global landscape of cell envelope protein complexes in *Escherichia coli*. *Nat. Biotechnol.* **36**, 103–112 (2018).

123 Malinverni, J. C. & Silhavy, T. J. An ABC transport system that maintains lipid asymmetry in the gram-negative outer membrane. *Proc. Natl. Acad. Sci. U.S.A.* **106**, 8009–8014 (2009).

124 Hughes, G. W. *et al.* Evidence for phospholipid export from the bacterial inner membrane by the Mla ABC transport system. *Nat. Microbiol.* **4**, 1692–1705 (2019).

125 Fisher, D. J., Fernández, R. E. & Maurelli, A. T. *Chlamydia trachomatis* transports NAD via the Npt1 ATP/ADP Translocase. *J. Bacteriol.* **195**, 3381–3386 (2013).

126 Schmitz-Esser, S. *et al.* ATP/ADP translocases: a Common Feature of Obligate Intracellular Amoebal Symbionts Related to Chlamydiae and Rickettsiae. *J. Bacteriol.* **186**, 683–691 (2004).

127 Trentmann, O., Horn, M., van Scheltinga, A. C. T., Neuhaus, H. E. & Haferkamp, I. Enlightening Energy Parasitism by Analysis of an ATP/ADP Transporter from Chlamydiae. *PLoS Biol.* **5**, e231 (2007).

128 Oswald, K. *et al.* Aerobic gammaproteobacterial methanotrophs mitigate methane emissions from oxic and anoxic lake waters. *Limnol. Oceanogr.* **61**, S101–S118 (2016).

129 Oswald, K. *et al.* Crenothrix are major methane consumers in stratified lakes. *ISME J* **11**, 2124–2140 (2017).

130 Takeshita, K. *et al.* Tripartite Symbiosis of an Anaerobic Scuticociliate with Two Hydrogenosome-Associated Endosymbionts, a *Holospora*-Related Alphaproteobacterium and a Methanogenic Archaeon. *Appl. Environ. Microbiol.* **85**, e00851-19 (2019).

131 Müller, M. The hydrogenosome. *J. Gen. Microbiol.* **139**, 2879-2889 (1993).

132 Hrdý, I., Tachezy, J. & Müller, M. Metabolism of Trichomonad Hydrogenosomes. in *Hydrogenosomes and Mitosomes: Mitochondria of Anaerobic Eukaryotes* (ed. Tachezy, J.) 127–158 (Springer Cham, 2019).

133 Lindmark, D. G. & Müller, M. Hydrogenosome, a cytoplasmic organelle of the anaerobic flagellate Tritrichomonas foetus, and its role in pyruvate metabolism. *J. Biol. Chem.* **248**, 7724–7728 (1973).

134 Nakazawa, M. *et al.* The origin of pyruvate: NADP+ oxidoreductase in mitochondria of *Euglena gracilis*. *FEBS Lett.* **479**, 155–156 (2000).
