# Supplementary material for: Anaerobic endosymbiont generates energy for ciliate host by denitrification
Source: Nature. 2021 Mar 3;591(7850):445–50. doi: 10.1038/s41586-021-03297-6 (PMC7969357; doi:10.1038/s41586-021-03297-6)
Supplement: Supplementary file 2 — Reporting Summary [file 41586_2021_3297_MOESM2_ESM.pdf]

nature research

Corresponding author(s): Jon Graf, Jana Milucka

Last updated by author(s): Jan 16, 2021

Reporting Summary

Nature Research wishes to improve the reproducibility of the work that we publish. This form provides structure for consistency and transparency in reporting. For further information on Nature Research policies, see Authors & Referees and the Editorial Policy Checklist.

Statistics

For all statistical analyses, confirm that the following items are present in the figure legend, table legend, main text, or Methods section.

n/a	Confirmed	
☐	☒	The exact sample size (*n*) for each experimental group/condition, given as a discrete number and unit of measurement
☐	☒	A statement on whether measurements were taken from distinct samples or whether the same sample was measured repeatedly
☐	☒	The statistical test(s) used AND whether they are one- or two-sided<br>*Only common tests should be described solely by name; describe more complex techniques in the Methods section.*
☒	☐	A description of all covariates tested
☒	☐	A description of any assumptions or corrections, such as tests of normality and adjustment for multiple comparisons
☐	☒	A full description of the statistical parameters including central tendency (e.g. means) or other basic estimates (e.g. regression coefficient) AND variation (e.g. standard deviation) or associated estimates of uncertainty (e.g. confidence intervals)
☒	☐	For null hypothesis testing, the test statistic (e.g. *F*, *t*, *r*) with confidence intervals, effect sizes, degrees of freedom and *P* value noted<br>*Give P values as exact values whenever suitable.*
☒	☐	For Bayesian analysis, information on the choice of priors and Markov chain Monte Carlo settings
☒	☐	For hierarchical and complex designs, identification of the appropriate level for tests and full reporting of outcomes
☒	☐	Estimates of effect sizes (e.g. Cohen's *d*, Pearson's *r*), indicating how they were calculated

Our web collection on statistics for biologists contains articles on many of the points above.

Software and code

Policy information about availability of computer code

Data collection

Data collection and analysis was performed using following software as detailed in the manuscript and supplementary information. ionOS 4.0.4, Sequencher 5.4.6, SINA aligner 1.2.11, Arb 6.1, MathFISH webserver (07/2017), Daime 2.1, Trimmomatic 0.32/0.39, metaSPAdes 3.13.0/3.9.1, SPAdes 3.13.3/3.10.1, oriFinder 1.0, Prokka 1.13.3, Pseudo finder 0.11, CDD 3.18, TransAAP (04/2020) , DNAplotter 18.1.0, eggNOG-mapper v1, MuscleWS 3.8.31, Jalview v2.11.1.0, SortMeRNA 2.1, Bowtie2 2.2.1.0, samtools 0.1.19, EDGE-pro 1.3.1, deepTools2 3.2.0, RNAmmer 1.5, RAxML 8.2.8, MAFFT online service v7, MAFFT 7.407, PhyloFlash 3.0/3.3b3, IQ-TREE 1.6.10/1.6.11, FigTree 1.4.4, iTOL 4, usearch 8.0.1623, MUSCLE 3.8.31, Bbmap 35.43, Matlab 2017b/2018b, Kaiju 1.7.3, rnaSPAdes 3.14.1, Transdecoder 5.5.0, BUSCO 4.0.6, MAFFT 7.407, FigTree 1.4.4, MAD 2.2, BLAST+ 2.9.0, MEGAN6 6.19.4, KEGG Automatic Annotation Server webserver (08/2020), HydDB webserver (08/2020), Zeiss ZEN 2.3, FEI xTM 6.2.6.3123

Data analysis

Data collection and analysis was performed using following software as detailed in the manuscript and supplementary information. ionOS 4.0.4, Sequencher 5.4.6, SINA aligner 1.2.11, Arb 6.1, MathFISH webserver (07/2017), Daime 2.1, Trimmomatic 0.32/0.39, metaSPAdes 3.13.0/3.9.1, SPAdes 3.13.3/3.10.1, oriFinder 1.0, Prokka 1.13.3, Pseudo finder 0.11, CDD 3.18, TransAAP (04/2020) , DNAplotter 18.1.0, eggNOG-mapper v1, MuscleWS 3.8.31, Jalview v2.11.1.0, SortMeRNA 2.1, Bowtie2 2.2.1.0, samtools 0.1.19, EDGE-pro 1.3.1, deepTools2 3.2.0, RNAmmer 1.5, RAxML 8.2.8, MAFFT online service v7, MAFFT 7.407, PhyloFlash 3.0/3.3b3, IQ-TREE 1.6.10/1.6.11, FigTree 1.4.4, iTOL 4, usearch 8.0.1623, MUSCLE 3.8.31, Bbmap 35.43, Matlab 2017b/2018b, Kaiju 1.7.3, rnaSPAdes 3.14.1, Transdecoder 5.5.0, BUSCO 4.0.6, MAFFT 7.407, FigTree 1.4.4, MAD 2.2, BLAST+ 2.9.0, MEGAN6 6.19.4, KEGG Automatic Annotation Server webserver (08/2020), HydDB webserver (08/2020), Zeiss ZEN 2.3, FEI xTM 6.2.6.3123

For manuscripts utilizing custom algorithms or software that are central to the research but not yet described in published literature, software must be made available to editors/reviewers. We strongly encourage code deposition in a community repository (e.g. GitHub). See the Nature Research guidelines for submitting code & software for further information.

Data

Policy information about availability of data

All manuscripts must include a data availability statement. This statement should provide the following information, where applicable:

- Accession codes, unique identifiers, or web links for publicly available datasets
- A list of figures that have associated raw data
- A description of any restrictions on data availability

The annotated genome of Ca. A. ciliaticola has been deposited at the European Nucleotide Archive (ENA) under the BioProject PRJEB27314 (accession number LR794158). Small subunit rRNA gene sequences of Ca. A. ciliaticola and the plagiopylean host have been deposited at ENA under BioProject PRJEB27314 and accession numbers LR798074-LR798089. Raw metagenomic and metatranscriptomic sequencing data of bulk water samples as well as single ciliate transcriptomes have been deposited at ENA under BioProject PRJEB36502 using the data brokerage service of the German Federation for Biological Data (GFBio). Metatranscriptomic sequencing data obtained in the year 2016 has been previously deposited at the Sequence Read Archive under BioProject PRJNA401219. Sequences used for phylogenetic trees are available under their respective accession codes at the SILVA rRNA database (http://www.arb-silva.de/; Fig. 2e, Extended Data Fig. 4), JGI Integrated Microbial Genomes and Microbiomes database (https://img.jgi.doe.gov/; Fig. 2e), EukRef-Ciliophora database (https://github.com/eukref/curation; Fig. 2f), orthoDB (https://www.orthodb.org/; Extended Data Fig. 5) or NCBI protein database (https://www.ncbi.nlm.nih.gov/protein/; Extended Data Fig. 8 and 9). Genomes used for comparative analyses are available under their respective accession codes (see Methods) at NCBI (https://www.ncbi.nlm.nih.gov/genome/; Fig. 2b, c). Transporter classification information (Supplementary Table 6) can be found at the Transporter Classification Database (https://www.tcdb.org/).

Field-specific reporting

Please select the one below that is the best fit for your research. If you are not sure, read the appropriate sections before making your selection.

☒ Life sciences ☐ Behavioural & social sciences ☐ Ecological, evolutionary & environmental sciences

For a reference copy of the document with all sections, see nature.com/documents/nr-reporting-summary-flat.pdf

Life sciences study design

All studies must disclose on these points even when the disclosure is negative.

Sample size	Sample size-based calculations were not relevant to this study and therefore no statistical methods were used to pre-determine sample size.
Data exclusions	No data were excluded from analysis.
Replication	Incubations experiments were performed in three independent biological replicates (bulk water samples) or two independent biological replicates (water enriched with ciliates, water without ciliates).<br>Metatranscriptomic analyses were performed separately using metatranscriptomic data obtained from two separate years (= independent biological replicates).<br>Genome reconstruction was independently performed twice using metagenomics data from separate two years (= independent biological replicates).<br>Please also refer to the Methods and Reproducibility section. All attempts at replication were successful. Similar results were obtained for all biological replicates.
Randomization	Randomization was not relevant to this study as it was not a randomized controlled trial involving participant groups.
Blinding	Blinding was not applicable because the study did not involve animals and/or human research participants. Furthermore, blinding was not possible because the person in charge of analyses was also responsible for taking the samples.

Reporting for specific materials, systems and methods

We require information from authors about some types of materials, experimental systems and methods used in many studies. Here, indicate whether each material, system or method listed is relevant to your study. If you are not sure if a list item applies to your research, read the appropriate section before selecting a response.

Materials & experimental systems

n/a	Involved in the study
☒	☐ Antibodies
☒	☐ Eukaryotic cell lines
☒	☐ Palaeontology
☒	☐ Animals and other organisms
☒	☐ Human research participants
☒	☐ Clinical data

Methods

n/a	Involved in the study
☒	☐ ChIP-seq
☒	☐ Flow cytometry
☒	☐ MRI-based neuroimaging
